# Drug‐Induced Periocular and Ocular Surface Disorders: An EAACI Position Paper

**DOI:** 10.1111/all.70074

**Published:** 2025-09-30

**Authors:** Andrea Leonardi, Banu Bozkurt, Diana Silva, Charlotte G. Mortz, Christophe Baudouin, Marina Atanaskovic‐Markovic, Vibha Sharma, Serge Doan, Shweta Agarwal, Daniel Pérez‐Formigo, Maria Joao Vasconcelos, Francoise‐Brignole Baudouin, Gonzalo Chorzepa, Jean‐Luc Fauquert, Virginia Calder, Pascal Demoly, Luis Delgado

**Affiliations:** ^1^ Ophthalmology Unit, Department of Neuroscience University of Padova Padova Italy; ^2^ Department of Ophthalmology Selçuk University Faculty of Medicine Konya Türkiye; ^3^ Basic and Clinical Immunology, Department of Pathology, Faculty of Medicine University of Porto Porto Portugal; ^4^ Immunoallergology Service, Department of Medicine ULS São João Porto Portugal; ^5^ Laboratory for Integrative and Translational Research in Population Health (ITR), EPIUnit—Institute of Public Health University of Porto Porto Portugal; ^6^ Departement of Dermatology and Allergy Center, Odense University Hospital University of Southern Denmark Odense Denmark; ^7^ Hôpital National de la Vision des 15‐20, INSERM‐DGOS CIC 1423 IHU FOReSIGHT Paris France; ^8^ University Paris Saclay, Versailles St Quentin Paris France; ^9^ Faculty of Medicine, University Children's Hospital of Belgrade University of Belgrade Belgrade Serbia; ^10^ Royal Manchester Children's Hospital Manchester UK; ^11^ Lydia Becker Institute of Immunology, and Inflammation University of Manchester Manchester UK; ^12^ Ophthalmology Fondation A de Rothschild and Hôpital Bichat Paris France; ^13^ CJ Shah Cornea Services/Dr G Sitalakshmi Memorial Clinic for Ocular Surface Disorders Medical Research Foundation Chennai Tamil Nadu India; ^14^ Department of Ophthalmology Hospital Universitario de Torrejon Madrid Spain; ^15^ Unidade de Imunoalergologia Hospital Lusíadas Porto, Lusíadas Saúde Porto Portugal; ^16^ Faculté de Pharmacie de Paris Université Paris Cité Paris France; ^17^ Sanatorio Parque Rosario Argentina; ^18^ Pediatric Allergy Unit, University Hospital Center Estaing Clermont‐Ferrand University Hospital Clermont‐Ferrand France; ^19^ UCL Institute of Ophthalmology London UK; ^20^ Division of Allergy, Department of Pulmonology, Allergy and Thoracic Oncology University Hospital of Montpellier Montpellier France; ^21^ Allergy Unit CUF Porto Hospital & Institute Porto Portugal; ^22^ RISE‐Health, Department of Pathology, Faculty of Medicine, Alameda Prof. Hernâni Monteiro University of Porto Porto Portugal

**Keywords:** biological treatments, drug related blepharoconjunctivitis, ocular allergy, ocular drug hypersensitivity reactions, severe cutaneous adverse reactions

## Abstract

Various systemic and topical medications can induce ocular and periocular cutaneous adverse effects (AEs), ranging from mild to severe. These AEs may lead to ocular surface (OS) damage and, in some cases, life‐threatening complications. Drug‐induced ocular adverse reactions are generally classified into two primary categories: toxic reactions and/or allergic hypersensitivity reactions, which can be IgE or non‐IgE‐mediated. Systemic antibiotics, antivirals, and anticonvulsants can trigger adverse reactions that may involve the OS. Drugs like antihistamines, beta‐blockers, antipsychotics, antidepressants, and isotretinoin are linked to dry eye disease. Topical treatments—including antibiotics, antiglaucoma medications, preservatives, contact lens solutions, and cosmetics—may elicit allergic or toxic ocular diseases. Recent evidence implicates ocular surface AEs in patients undergoing biological treatments for oncological diseases and atopic dermatitis. Epidermal growth factor receptor inhibitors, used in the treatment of several cancers, have been associated with conjunctivitis, meibomitis, dry eye, periocular skin changes, and trichomegaly. Similarly, dupilumab, the first biologic approved for treating moderate‐to‐severe atopic dermatitis, has also been linked to OS disease with blepharoconjunctivitis. This position paper provides a comprehensive overview of the clinical presentations, diagnostic approaches, and treatment strategies for drug‐induced ocular AEs, integrating the latest literature and clinical guidelines.

## Graphical abstract

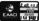

## 1 Introduction

**FIGURE 1:**
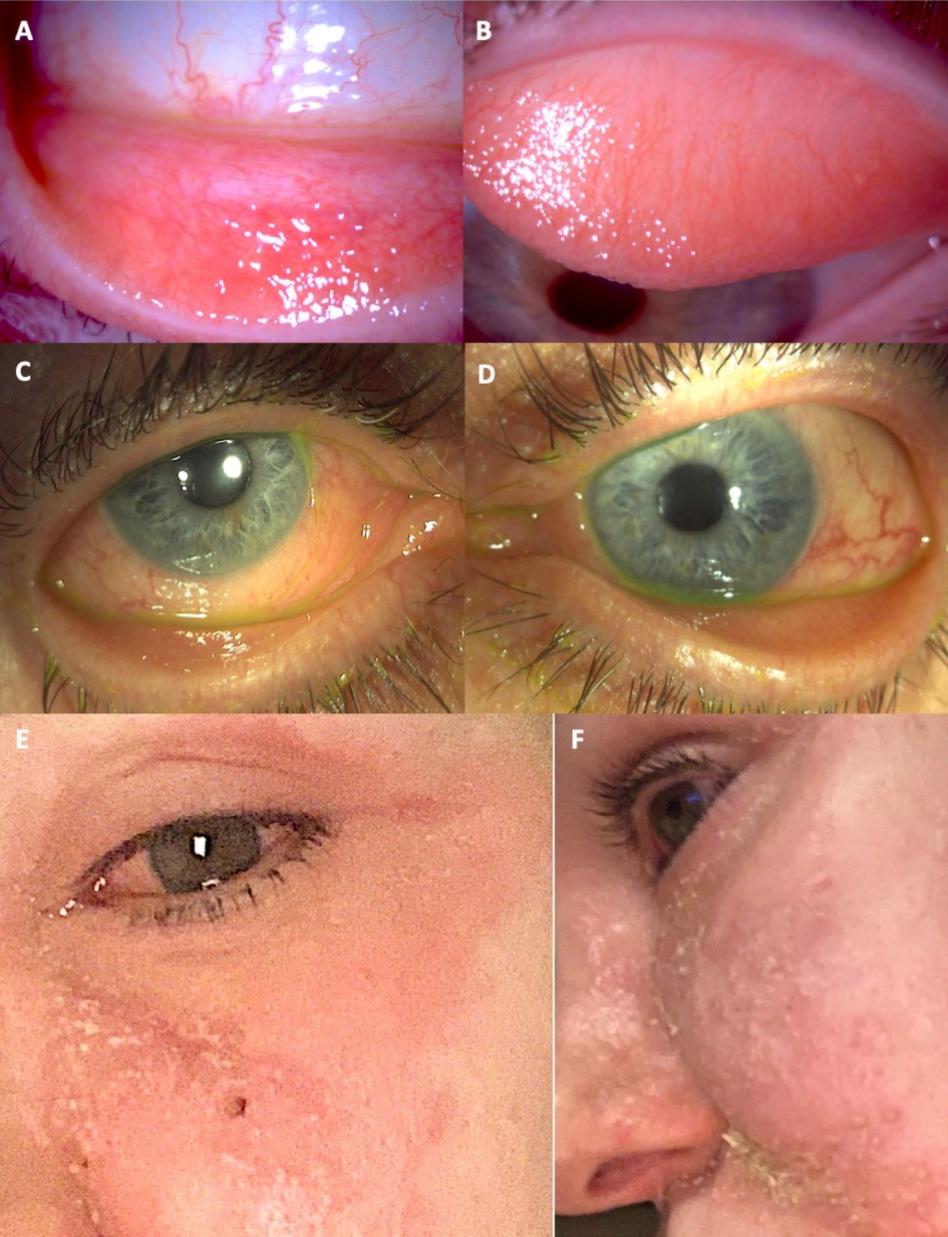
Different phenotypes of ocular drug hypersensitivity reactions (ODHR). (A) Lower and (B) upper lid conjunctival follicular reaction do to topical drugs. (C, D) Bilateral chronic ODHR. (E, F) Periocular and facial eczematous reaction as ODHR.

With the increasing global exposure and prolonged use of medications, there has been a corresponding rise in the risk of developing drug hypersensitivity reactions (DHRs) [1, 2], including those affecting the eye and the ocular surface (OS). DHRs are known to affect approximately 7% of the general population. However, drug‐related ocular reactions remain poorly defined regarding their epidemiology, phenotypes, and endotypes. The Ocular Allergy Working Group (OAWG) previously classified ocular allergies as either IgE‐mediated or non‐IgE‐mediated diseases, without specifying a classification for ocular drug reactions [3]. In light of recent international consensus on drug allergies and updated classification of cutaneous DHRs [2], we propose adopting the term “ocular DHRs” (ODHRs) to describe objectively reproducible ocular symptoms or signs initiated by exposure to a defined drug, at a dose typically tolerated by a normal individual, and which clinically resemble allergic reactions [2]. ODHRs may present with distinct phenotypes, variable onset, and severity (Figure 1). These reactions can be categorized as:

1. Periocular (cutaneous)/eyelid hypersensitivity reactions: this category encompasses both IgE‐ and non‐IgE‐mediated responses, manifesting as eyelid urticaria, hyper‐acute eyelid edema, with or without conjunctival swelling.
2. Delayed reactions: these involve the skin of the eyelid, the lid margin, and/or the conjunctiva, potentially leading to eyelid exanthemas/eczema, blepharitis, follicular conjunctivitis, cicatrizing conjunctivitis, and ocular pseudo‐pemphigoid.

The cornea may be indirectly affected by the release of epithelial‐toxic mediators, lid margin abnormalities, limbal cell deficiency, or cicatrizing phenomena. In some cases, direct corneal involvement may occur through immune‐complex deposition.

Different factors contribute to the risk of ODHRs including drug‐related factors, genetic predisposition, comorbidities, and local factors. Both topical agents (such as over‐the‐counter eye drops, antiglaucoma drugs, antibiotics, eyedrop preservatives, ointments, moisturizers, disinfectants, contact lens solutions, cosmetics) and systemic medications have the potential to induce various phenotypes through mechanisms such as IgE‐ and T‐cell mediated responses, local toxicity due to nonimmune cell‐receptor interactions, immune complex‐mediated reactions, and cytotoxic IgG‐mediated reactions [4, 5, 6].

The most severe forms of ocular involvement may occur in drug‐induced severe cutaneous adverse reactions (SCARs) including Stevens‐Johnson syndrome (SJS), toxic epidermal necrolysis (TEN), and SJS/TEN overlaps. New phenotypes such as the dupilumab‐induced ocular surface disease (DIOSD) [7] and ocular AEs associated with newer biological treatments have emerged.

This paper aims to provide an updated overview of the clinical features, mechanisms, diagnostic methods, and treatment options in ODHRs. We aim to support ophthalmologists, allergists, dermatologists, pediatricians, and internists who frequently prescribe these drugs or manage related complications.

## 2 Materials and Methods

With this paper, the Task Force on “Drug‐Induced Periocular and Ocular Surface Disorders” approved by the European Academy of Allergy and Clinical Immunology (EAACI) provides an expert‐driven synthesis rather than guideline‐level recommendations, based on a combination of published evidence and expert consensus. A comprehensive literature search was performed using PubMed and Medline (supplementary methods section). Given the heterogeneity of the topics and the low quality of evidence, and since the manuscript aimed to synthesize current evidence and raise awareness rather than provide formal guidance, grading was not conducted. The development of this position paper included several rounds of consultation through virtual and in‐person meetings. Position statements were formulated for each chapter and voted on. Due to the limited availability of high‐quality evidence, the statements represent an expert consensus.

## 3 Systemic Medications and Ocular Surface Disorders

The interaction between systemic medications and ocular health has become an increasingly important focus in clinical practice. Many commonly prescribed drugs are associated with a wide range of ocular adverse effects (AEs), some of which may result in permanent visual loss. Twenty‐two out of the 100 most frequently used systemic drugs in the United States have the potential to cause dry eye disease (DED) [8]. Drug molecules can accumulate in the cornea, lens, and retina, leading to a variety of symptoms associated with drug toxicity [9]. While many ocular AEs caused by systemic medications are reversible, delayed detection and management may result in irreversible ocular damage and visual impairment. Early recognition and appropriate treatment are crucial in preventing long‐term complications.

### 3.1 Factors Increasing Risk of Ocular Complications

Pre‐existing conditions such as end‐stage renal disease, liver disease, diabetes, pregnancy, or glaucoma represent an increased risk for ocular damage; therefore, systemic medications may further exacerbate these conditions. Clinicians must be aware of these risks and closely monitor patients to facilitate the early detection and treatment of ocular complications. Age is a risk factor for DED because of a reduced basal tear production and potentially decreased drug clearance rates. The typical use of multiple medications in older subjects may increase the susceptibility to ocular AEs [10]. However, it may be challenging to determine whether ocular pathology is due to the underlying disease or the medication used to treat it.

### 3.2 Mechanisms of Systemic Drug‐Induced Ocular Disorders (Table 1)

**TABLE 1:** Mechanisms of ocular adverse effects induced by systemic drugs.

Mechanism	Examples of drugs or drug families	Clinical manifestations
1. Reduced tear production: Systemic drugs with anticholinergic effects inhibit parasympathetic stimulation of the lacrimal glands, reducing tear production and increasing hyperosmolarity of the tear films, which incites ocular inflammation	Antihistamines, antidepressants (e.g., tricyclic antidepressants, selective serotonin reuptake inhibitors), β‐blockers, diuretics, corticosteroids	Dry eye disease: Dryness, burning, itching, foreign body sensation, and fluctuating vision worsened by prolonged visual tasks (e.g., reading or screen use) Keratoconjunctivitis sicca: A severe form of dry eye causing corneal/conjunctival damage, with photophobia, hyperemia, and potential corneal ulceration
2. Altered tear film composition: Some medications alter the lipid or mucin layers of the tear film, causing instability with increased tear evaporation and less lubrification	Isotretinoin, hormone replacement therapy, oral contraceptives, and certain antipsychotics	Evaporative dry eye: Excessive tearing,^a^ dryness, foreign body sensation and intermittent blurred vision, which clears with blinking Meibomian gland dysfunction: Drugs like isotretinoin can affect the meibomian glands, causing thickened or absent meibum secretion, leading to gland blockage, inflammation and worsening of evaporative dry eye
3. Direct toxicity: Some drugs directly damage corneal or conjunctival epithelial cells, disrupting integrity and causing inflammation, cell death, or ulceration	Chemotherapy agents (5‐Fluoro uracil), antivirals (cidofovir), anti‐tuberculous, antimalarial, antiglaucoma (β‐blockers, prostaglandins), bisphosphonates	Corneal erosions or ulcers: Sharp pain, photophobia, decreased vision acuity, with superficial punctate keratitis or severe ulceration Conjunctivitis: Red, irritated eyes with burning, itching, discharge, or conjunctival swelling
4. Inflammatory responses: Certain drugs trigger immune‐mediated inflammation of the conjunctival or cornea through hypersensitivity or immune dysregulation	Nonsteroidal anti‐inflammatory drugs, immunomodulators (tumor necrosis factor‐α inhibitors, corticosteroids), antibiotics (sulfonamides, beta lactams), alopurinol, antiepileptics	Allergic conjunctivitis: Redness, itching, watery discharge, eyelid swelling, and potential conjunctival scarring if persistent Keratitis: Painful, red eyes with gritty sensation; untreated cases may lead to corneal ulcers and vision loss Stevens‐Johnson syndrome: Severe drug reaction causing extensive ocular damage, conjunctivitis, corneal scarring, and possible blindness
5. Altered blood flow: Some medications affect ocular blood vessels, causing ischemia or altered permeability, leading to decreased nourishment of the ocular surface, tissue damage, and inflammation	Vasoconstrictive agents, systemic beta‐blockers, and chemotherapeutic agents	Conjunctival ischemia: Pale conjunctiva, discomfort, dryness, or grittiness Corneal neovascularization: Abnormal blood vessel growth in the cornea causing blurred vision and scarring
6. Impact on tear gland function: Some medications cause structural damage or dysfunction in lacrimal glands, leading to a long‐term decrease in tear production due to gland toxicity or fibrosis following chronic use of these drugs	Chemotherapy agents, radiotherapy, and immunosuppressive drugs	Chronic dry eye disease: Persistent dry eye symptoms resistant to standard treatments due to irreversible gland damage. Patients may need long‐term use of artificial tears, punctal plugs, or other therapies

^a^ Paradoxical reflex tearing due to eye irritation: excess tears temporarily restore the tear film, but evaporation soon exceeds basal production, leading to repeated tearing.

Some drugs are known to cause specific AEs such as corneal deposits in patients taking amiodarone or tamsulosin‐induced intraoperative floppy iris syndrome [9, 11, 12, 13]. Corticosteroids can reduce tear production and alter the immune response, increasing the risk of ocular infections. While topical and periocular corticosteroids carry the highest risk for cataracts and glaucoma, systemic corticosteroids can have similar effects, particularly when used at moderate to high doses over extended periods [14]. Although no formal screening guidelines exist, routine ocular evaluations are recommended for patients with chronic corticosteroid treatment.

### 3.3 Diagnosis and Management

A thorough patient history, including current and past medication use, is essential for establishing potential links between drug use and ocular surface disease (OSD). In the suspect of an ODHR, dose adjustment, drug discontinuation, or switch to a safer alternative is recommended. The management of DED involves artificial tears, lubricating ointments, and punctal plugs. Autologous serum eye drops or anti‐inflammatory drugs like cyclosporine or lifitegrast may be necessary in more severe cases. Warm compresses, lid hygiene, and oral omega‐3 fatty acids may be effective in meibomian gland dysfunction (MGD). If isotretinoin is the cause of MGD, dose adjustment or drug discontinuation is necessary. Topical corticosteroids or nonsteroidal anti‐inflammatory drugs (NSAIDs) can help to manage OSDs, but should be used with caution because of the risk of inducing other AEs.

### 3.4 Screening and Collaborative Care

For certain medications, regular screening protocols have already been established. For instance, due to the risk of retinal toxicity, the American Academy of Ophthalmology recommended screening for hydroxychloroquine [15]. Regular ophthalmologic evaluation is recommended every two months in the case of ethambutol treatment [16], every 6–12 months to detect early signs of cataract and increased intraocular pressure corticosteroids‐induced.

## 4 Severe Cutaneous Adverse Reactions (SCARs) and the Ocular Surface

Among drug‐related SCARs, SJS and TEN are most commonly associated with ocular involvement. Eyelid blisters and conjunctivitis have also been occasionally reported in drug reaction with eosinophilia and systemic symptoms (DRESS), generalized bullous fixed drug eruption (GBFDE), and acute generalized exanthematous pustulosis (AGEP) [17]. Risk factors include drug‐related factors (specific medications), individual genetic predisposition (HLA allele variations), co‐morbidities (underlying health conditions), and local factors (environmental influences) [4]. NSAIDs, anti‐epileptic drugs, sulfonamide antibiotics, and allopurinol are major triggers of SCARs, some of which are associated with specific HLA class I alleles such as HLA‐B*58:01, HLA‐B*13:01, and HLA‐B*57:01 [18, 19]. In SJS/TEN, HLA‐mediated drug presentation activates oligoclonal CD8+ cytotoxic T cells [20]. However, HLA alleles alone are insufficient; granulysin, TNF‐α, mitochondrial apoptosis [21], neutrophil extracellular traps [22], and necroptosis also contribute to keratinocyte death [20, 21, 23]. Impaired T‐regulatory cell function [24] and JAK/STAT pathway activation are further potential mechanisms and therapeutic targets [25].

The severity‐of‐illness score for SJS and TEN (SCORTEN) does not correlate with ocular complications, suggesting that the clinical and pathogenic connections between the OS and skin involvement in SCARs remain poorly understood [17].

### 4.1 Ophthalmic Manifestations in SJS/TEN

#### 4.1.1 Acute Phase

**FIGURE 2:**
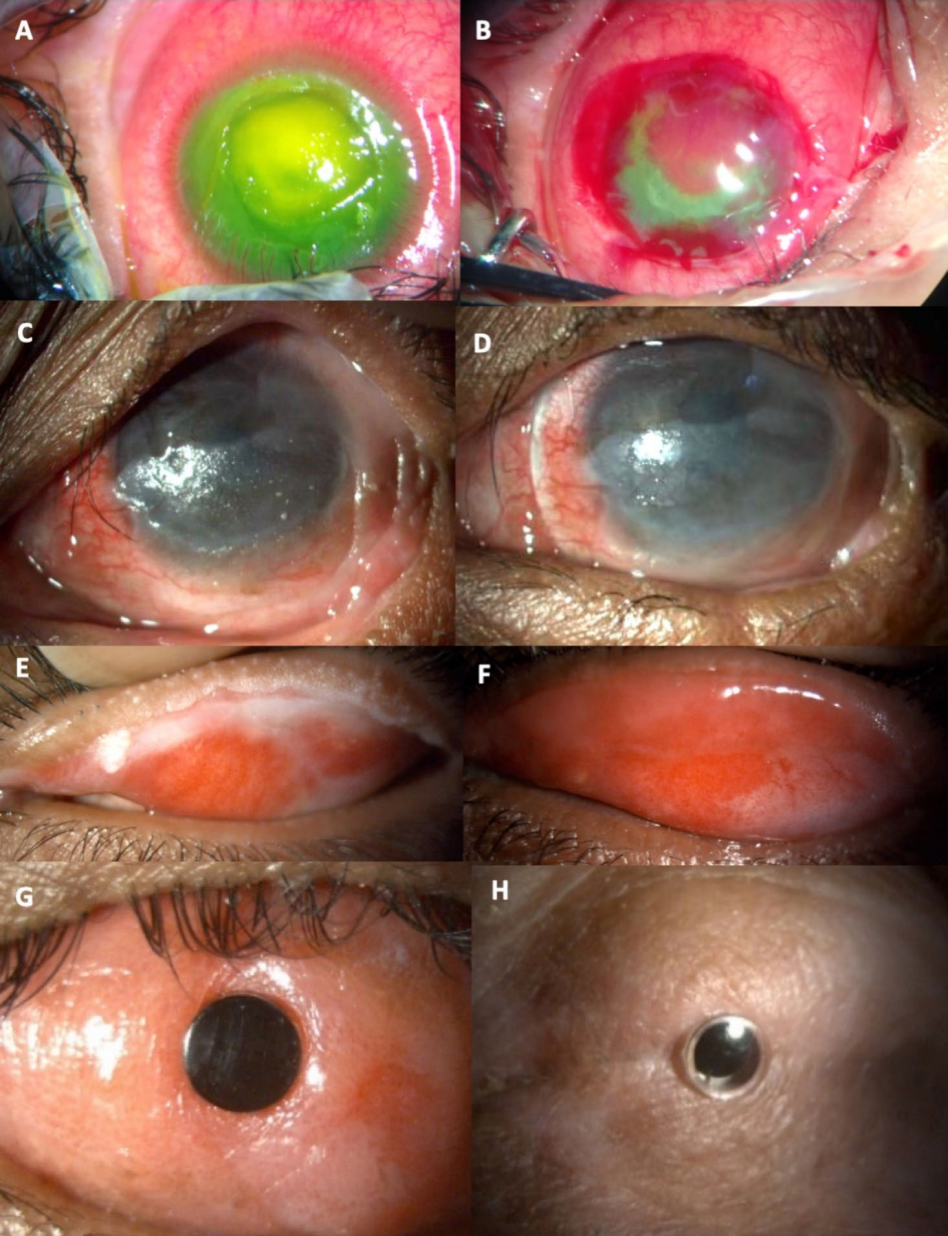
Acute a chronic phase of SJS. (A) An acute SJS patient with severe inflammation and a persistent corneal defect. (B) The same patient treated with amniotic membrane graft done using perilimbal purse string sutures. (C) SJS patient in the chronic ocular surface inflammatory phase with dry keratinized surface with minimal haze and low visual function. (D) The same patient with decreased inflammation and visual improvement after a scleral contact lens application. (E) Lid margin keratinization along the upper lid. (F) Keratinized tissue replaced by oral mucous membrane graft. (G) Modified osteodonto keratoprosthesis (MOOKP) in one SJS patient. (H) Boston type 2 keratoprosthesis in one SJS patient.

Ocular involvement may range from conjunctival hyperemia to near‐total conjunctival and corneal epithelial defects. Approximately 50%–80% of cases exhibit ocular manifestations including bilateral mucopurulent conjunctivitis, inflammatory pseudomembranes, early symblepharon formation, punctate epithelial keratitis, corneal ulceration, and corneal perforation [17, 26, 27] (Figure 2A,B). Eyelid margin involvement is also common, presenting as meibomitis and/or epithelial sloughing [26, 27].

#### 4.1.2 Chronic Phase

The severity of chronic complications depends on the extent of acute phase involvement. Inflammation and ulceration of the conjunctiva result in scarring and symblepharon in 41%–71% of patients, severe DED in 45%–56% [28, 29]. Conjunctival scarring reduces goblet cell density, while lacrimal duct fibrosis leads to aqueous DED. Meibomian gland involvement further exacerbates tear film dysfunction. Eyelid abnormalities include entropion, ectropion, trichiasis, distichiasis, and punctal stenosis. Lid margin keratinization, a hallmark feature of SJS, induces blink‐related microtrauma [17, 27], resulting in chronic inflammation, repeated corneal abrasions, scarring, vascularization, and limbal stem cell deficiency, leading to corneal blindness [30].

### 4.2 Management

A treatment algorithm emphasizing the concept of a “window of opportunity” has been suggested, highlighting the importance of early and regular ophthalmic examinations tailored to the disease stage and specific ocular findings [27].

#### 4.2.1 Acute Care

Ophthalmologic evaluation, including fluorescein staining, should begin in the Intensive Care Unit within the first 2–6 days after onset and continue throughout the resolution of skin and mucosal ulcerations [27]. For patients with conjunctival hyperemia without epithelial defects, treatment includes topical antibiotics, corticosteroids, and intensive lubrication. In cases with epithelial defects involving the conjunctiva, cornea, or lid margin, early amniotic membrane transplantation (AMT) has shown favorable outcomes [31, 32], due to its immunomodulatory and anti‐inflammatory properties that promote epithelial healing. Topical corticosteroids, used cautiously in the presence of epithelial defects, help control inflammation and prevent further OS breakdown. In corneal epithelial defects, bandage contact lenses may be useful (Figure 2C,D). Saline rinses, frequent lubrication, and pseudomembrane debridement are also recommended [27, 31, 32]. Ensuring adequate lid closure is critical, as lagophthalmos and corneal exposure can exacerbate OS damage.

There is little consensus on the optimal drug management and evidence‐based recommendations of SJS and TEN acute phases [33]. The use of anti‐TNFα has been associated with lower mortality compared to corticosteroids. The effectiveness of intravenous immunoglobulin and cyclosporine remains uncertain [34]. Since the JAK/STAT pathway was identified as a potential therapeutic target, a short course of JAK inhibitors significantly improved TEN patients without AEs or mortality [25].

#### 4.2.2 Chronic Care

Management of the chronic phase focuses on OS stabilization and visual rehabilitation.

*(a) Ocular surface stabilization*. Punctal cautery combined with intensive lubrication has been effective in stabilizing the OS and improving tear film in severe DED [35]. Mucous membrane grafting can reduce blink‐related microtrauma from the lid margin keratinization, improving OS integrity and vision (Figure 2E,F) [35, 36]. Timely correction of trichiasis, entropion, and ectropion is essential to prevent ongoing inflammation and OS breakdown [35, 36]. Short courses of topical antibiotics may also help control recurrent inflammation by addressing the altered ocular microbiome [37]. Systemic immunomodulators have not consistently improved visual outcomes or ocular complications and remain controversial [38, 39]. Desensitization to the culprit drug is strongly contraindicated in SJS, TEN, and DRESS by the EAACI Drug Hypersensitivity Interest Group [40], despite isolated reports of success in sulfamethoxazole‐induced SJS cases [41].

*(b) Visual rehabilitative procedures* should only be considered once the OS is stabilized to reduce postoperative complications. Due to the underlying immune dysfunction often associated with DED and eyelid abnormalities, corneal and limbal stem cell transplants carry a high risk of rejection and are generally not recommended. Cataract surgery is always a challenge because of corneal haze, vascularization, and fornix shortening. Visual outcomes can be improved with scleral lenses in eyes with partially scarred or keratinized corneas [35]. In patients with end‐stage OSD, keratoprosthesis remains the only viable option [42, 43, 44, 45] (Table S1) (Figure 2G,H).

## 5 Ocular Adverse Effects of Biological Treatments

Biological agents, including monoclonal antibodies (mAB), cytokine inhibitors, and other targeted immunotherapies, have been associated with ocular AEs, which may occur directly or indirectly through systemic immune modulation. The most reported ocular complications include DED, OSD, uveitis, optic neuropathy, and retinal toxicity.

### 5.1 TNFα Inhibitors

TNFα inhibitors are mAB that function by competitively preventing TNFα from binding its receptors. Etanercept, infliximab, adalimumab, certolizumab pegol, and golimumab have been approved by the FDA for the treatment of rheumatoid arthritis (RA), inflammatory bowel disease, ankylosing spondylitis, psoriasis, and Behçet's disease. Although TNFα inhibitors are generally well tolerated and typically associated with minor side effects, several serious AEs have been reported. These include infections (reactivation of latent tuberculosis), lymphomas, congestive heart failure, cytopenia, demyelinating disorders, lupus‐like syndromes, and induction of autoantibodies. Anterior uveitis has been identified as the most common ocular AE. Of the abovementioned TNFα inhibitors, etanercept is the most likely of this class to cause drug‐induced uveitis [46]. Only a limited number of cases described peripheral corneal infiltrates, cicatrizing conjunctivitis, and severe blepharitis with ectropion associated with adalimumab [47, 48, 49, 50], which regressed following the drug discontinuation.

### 5.2 IL‐6 Inhibitors

Tocilizumab (TCZ), a humanized mAB targeting both soluble and membrane‐bound IL‐6, has been approved for the treatment of RA and both polyarticular and systemic juvenile idiopathic arthritis (JIA) [51]. It is effective in 76% of patients with severe JIA‐associated uveitis unresponsive to conventional immunosuppressive therapies [52]. AEs include viral conjunctivitis with bullous impetigo [53] and peripheral ulcerative keratitis (PUK), a rare and destructive inflammatory corneal disease [54, 55, 56, 57].

### 5.3 Anti‐IL4/IL‐13R

Dupilumab, a mAB inhibiting IL‐4 and IL‐13 signaling pathways, is approved for the treatment of moderate‐to‐severe atopic dermatitis (AD), asthma, chronic rhinosinusitis with nasal polyposis, and eosinophilic esophagitis. In patients with AD, dupilumab has been associated with a specific ocular AE named DIOSD or dupilumab‐associated OSD (DAOSD), with blepharoconjunctivitis as a common clinical manifestation [58].

#### 5.3.1 Factors Increasing the Risk of Ocular AEs

AD patients are already at an increased risk of developing OSD [59]. DIOSD is among the most frequently reported AEs in real‐world studies involving dupilumab‐treated AD patients [58] with a much higher incidence than in patients treated for other indications [60, 61]. In randomized controlled trials (RCTs), the incidence of DIOSD among AD patients ranged from 8% to 22% [60], while prospective observational studies reported higher incidences (19%–32%) [62, 63]. In contrast, tralokinumab (anti‐IL‐13 mAB) has been associated with lower rates of ocular AEs (2%–13%) [64]. Risk factors for DIOSD are history of allergic/atopic conjunctivitis and blepharitis [62], “any other eye disease” and concurrent use of topical ocular treatments [62]. In a prospective study, including baseline ophthalmological assessment, DED was the only significant ocular risk factor [63]. Other risk factors include severe AD, periocular eczema, erythroderma, elevated serum levels of IgE, and of Thymus and activation‐regulated chemokine (TARC/CCL17) [65].

#### 5.3.2 Mechanisms of Ocular AEs and Manifestations

The exact pathophysiology of DIOSD is unclear. The prevailing hypothesis involves the imbalance between Th2 and Th1/Th17 pathways, as evidenced by the reported tear cytokine profiles in AD patients with and without DIOSD [66]. IL‐13‐mediated conjunctival goblet cell dysfunction may also contribute to decreased mucin production and tear film homeostasis [67]. Other proposed mechanisms include superinfection with *Demodex* mites, OX40 ligand activation, and goblet cell depletion, suggesting similarities between DIOSD and DED [7].

#### 5.3.3 Clinical Manifestations

**FIGURE 3:**
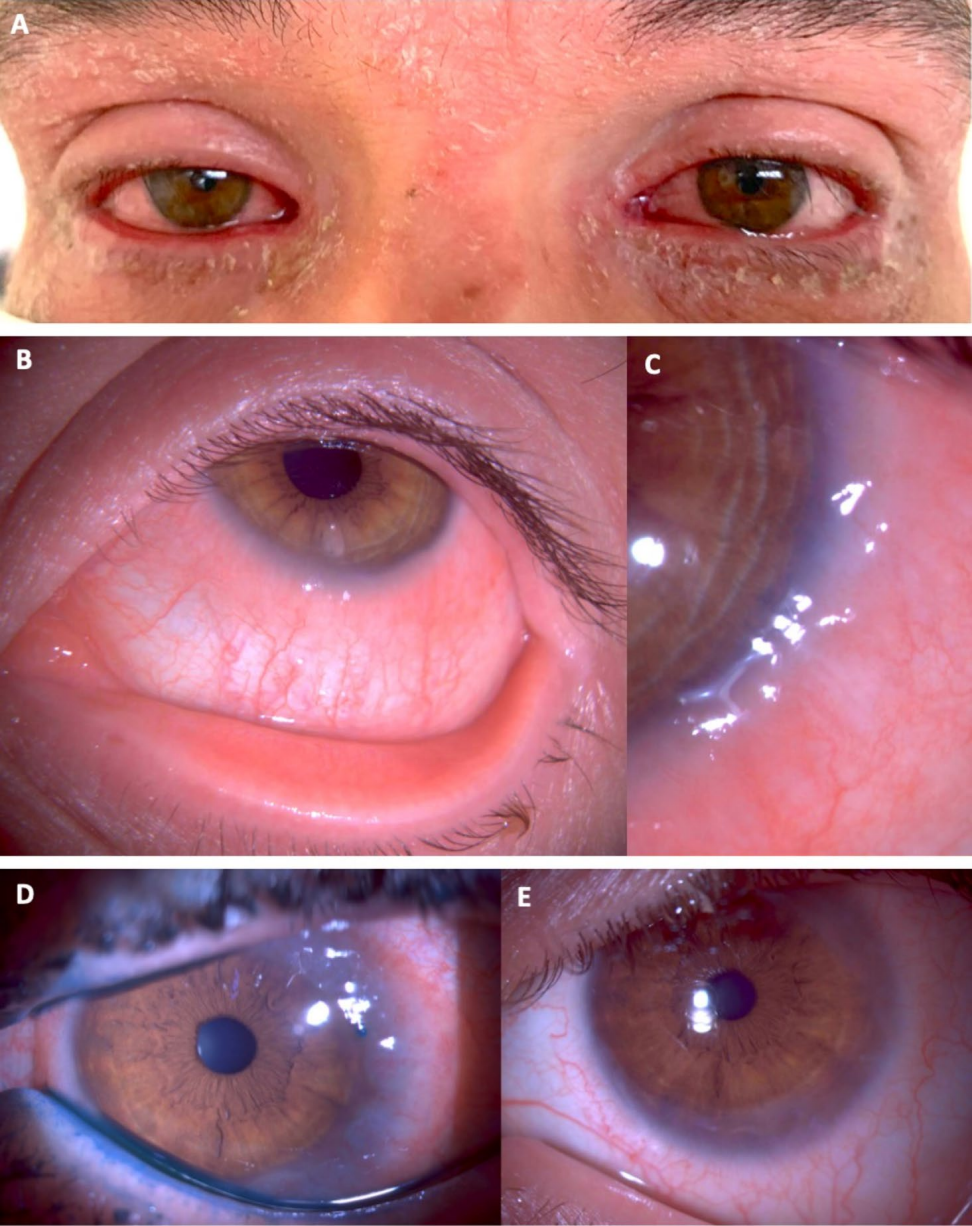
Dupilumab induced ocular surface disease (DIODS). (A) Typical blepharoconjunctivitis in a DIODS patient. (B) Severe conjunctival inflammation in a DIODS patient. (C) Note the severe limbal involvement at a higher magnification in the same patient. (D) A DIODS patient with severe conjunctival, limbal, and peripheral corneal involvement (the lissamine green stain shows the lid margin involvement) successfully treated with topical tacrolimus 0.1% compounded eyedrops (E).

DIOSD closely resembles atopic keratoconjunctivitis (AKC) with less severe corneal involvement. Signs and symptoms include ocular and periocular itching and burning, dryness, mucous discharge, conjunctival hyperemia, palpebral papillary hypertrophy, limbal inflammation with Trantas‐Horner's dots, MGD and evaporative DED, lid margin blepharitis, and eyelid dermatitis (Figure 3A–C). Corneal involvement typically manifests as superficial punctate keratopathy. The severity and presentation can vary significantly between individuals, ranging from mild DED symptoms to isolated periocular dermatitis, conjunctival inflammation, or a combination of all signs. While symptoms may improve over time (usually months), inflammatory flares can occur following dupilumab administration. Conjunctival scarring has been rarely reported. Although chronic ocular symptoms can affect quality of life (QoL), long‐term prognosis is generally favorable, with uncommon corneal complications.

#### 5.3.4 Management

Eyelid dermatitis is typically managed with emollients, topical corticosteroids, and calcineurin inhibitors (tacrolimus ointment) [68]. Ocular involvement requires artificial tears, antihistamine or mast cell stabilizer eye drops, and eyelid hygiene (warm compresses and massage). Short‐term use of ocular topical corticosteroids may be necessary during acute exacerbations. In more persistent/severe cases, cyclosporine or tacrolimus eye drops may be useful (Figure 3D,E). In refractory cases, therapeutic strategies include extending the interval between dupilumab injections or switching to alternative biologics such as tralokinumab or JAK inhibitors [69]. There are no data on re‐introduction of dupilumab after its discontinuation due to uncontrolled DIOSD. In real‐world practice, most specialists prefer to switch to another treatment and not risk a recurrence.

## 6 Ocular Adverse Effects in Cancer‐Targeted Therapy

Unlike conventional chemotherapy, cancer‐targeted agents (CTAs) selectively disrupt molecular pathways crucial for tumor growth and metastasis, often through mABs and antibody‐cytokine fusion proteins [70, 71, 72]. While these therapies reduce systemic toxicity, they can cause ocular AEs needing prompt recognition and management [73, 74, 75, 76, 77, 78] (Table S2). The incidence and severity of ocular AEs depend on the specific agent, dosage, and treatment duration. Mechanistically, these effects may result from disruption in eyelash follicular cycling, immune system overactivation, elevated levels of inflammatory cytokines, disruption in the maintenance of interstitial pressure, deposition of therapeutic agents within the corneal epithelium, and off‐target toxicity.

### 6.1 Signal Transduction Inhibitors (Table S3)

**FIGURE 4:**
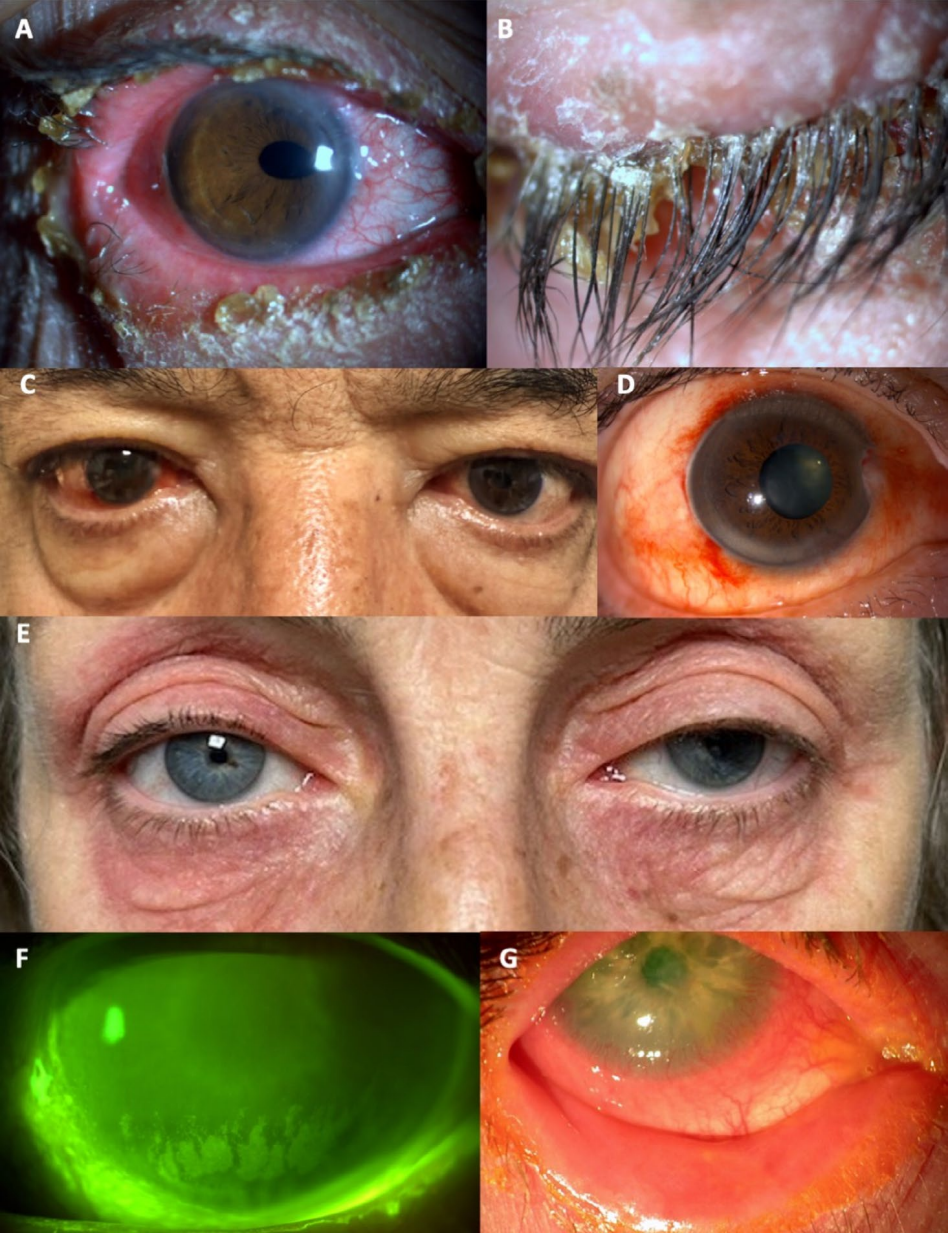
(A) Ulcerative blepharitis with crusty secretion at the lid margin, conjunctivitis and trichomegaly (B) induced by EGFR tyrosine kinase inhibitors. (C) PDGFR TK1 (imatinib)‐induced periorbital edema and (D) subconjunctival hemorrhages. (E) Contact eyelid dermatitis induced by cosmetics. (F) Toxic keratopathy with partial hyposensitivity in a glaucoma patient with little complaint despite significant superficial punctate epitheliopathy highlighted by fluorescein staining and yellow filter. (G) Severe blepharokeratoconjunctivits in a patient treated with topical antiglaucoma drugs.

Epidermal growth factor receptor inhibitors (EGFRI) are widely used in treating solid tumors including non‐small cell lung, colorectal, head and neck, breast, and pancreatic cancers [79]. Two main classes exist: mAB and tyrosine kinase inhibitors (TKI). Reported ocular surface AEs are trichomegaly, trichiasis, blepharitis, MGD, DED, conjunctivitis, and keratitis (Figure 4A,B) [80, 81, 82, 83, 84, 85, 86, 87, 88, 89, 90, 91, 92, 93, 94, 95, 96]. Inhibition of the EGFR signaling in the hair follicle sheath disrupts the normal hair follicle growth cycle, leading to eyelash changes. Suppression of corneal epithelial cell proliferation impairs tissue regeneration and healing, increasing susceptibility to environmental insults (e.g., dryness, particulate matter).

BCR/ABL, c‐kit, and PDGFR TKI (Imatinib) are used in the treatment of chronic myeloid leukemia and gastrointestinal stromal tumors [73]. Common ocular AEs are periorbital edema (up to 70%), epiphora (20%), and conjunctival hemorrhage (11%) (Figure 4C,D) [73, 97, 98, 99, 100, 101, 102]. Periorbital edema is attributed to dermal dendrocytes in the periocular soft tissue, which express imatinib targets such as c‐kit and PDGFR. By inhibiting PDGFR, imatinib may reduce interstitial pressure and enhance trans‐capillary fluid transport.

Multitarget TKI vandetanib inhibits EGFR, VEGFR, and the RET protooncogene and is used in rare cases of advanced medullary thyroid cancers. Its accumulation in the basal corneal epithelium has been associated with vortex keratopathy [95].

Erdafitinib, *a TKI of FGFRs 1 to 4*, is indicated for the treatment of locally advanced, unresectable, or metastatic urothelial carcinoma. Reported ocular surface AEs are DED, conjunctivitis, keratitis, cataracts, trichiasis, corneal ulceration, and MGD [103].

Perifosine, *a PI3K/Akt/mTOR inhibitor*, is used in colorectal cancer and multiple myeloma and was shown to cause peripheral, ring‐shaped, superficial corneal stromal infiltration and ulcers resembling autoimmune keratitis, typically responsive to topical corticosteroids [104, 105].

### 6.2 Immune Checkpoint Inhibitors (ICIs) (Table S4)

ICIs are mAB targeting CTLA‐4, programmed‐death protein‐1 (PD‐1), its ligand PD‐L1, and LAG‐3 [70, 71, 72, 74, 75]. Ocular AEs occur in approximately 1% of patients within weeks to months of therapy initiation [106]. The most commonly reported AEs are DED, conjunctivitis, and corneal pathologies, especially in patients treated with PD1/PD1‐L inhibitors [107, 108, 109]. DED appears to result from the production of autoantibodies targeting the lacrimal gland, as well as sarcoid‐like granulomatous inflammation driven by CD8+ T‐cell infiltration and IL‐2 production. ICIs can induce persistent corneal epithelial defects, corneal melting, and perforation, attributed to immune checkpoint dysregulation on the cornea, leading to uncontrolled T‐cell hyperactivity, inflammatory cytokine release, autoantibody production, immune complex deposition, stromal inflammation, and keratolysis [71, 74, 110, 111, 112, 113, 114, 115, 116, 117, 118, 119]. Additionally, ICIs increase the risk of solid organ and corneal graft rejection (20%–40%) [120, 121].

### 6.3 Antibody‐Drug Conjugates (ADC) (Table S5)

ADCs combining mAB with cytotoxic agents (payload) via chemical linkers are approved for the treatment of various solid and hematological cancers [122]. Ocular AEs include DED, conjunctivitis, and corneal abnormalities (microcyst‐like corneal epithelial cysts (MEC), superficial punctate keratitis, and keratoconjunctivitis, limbal stem cell deficiency, and neuropathy) [77, 123, 124, 125, 126]. These AEs are largely attributed to off‐target toxicity. Proposed mechanisms include suboptimal linker stability, receptor‐mediated and nonspecific endocytosis, as well as the bystander effect [127].

### 6.4 Management

A baseline ophthalmologic examination is recommended before initiating CTAs. Most AEs are often mild and reversible with dose adjustment. EGFRI‐induced eyelash changes can be managed with lid hygiene, lash trimming, lubricants, topical antibiotics, or immunomodulators. Severe cases may necessitate bandage contact lenses, electrolysis, laser treatment, cryotherapy, or surgical excision of the abnormal eyelashes. DED symptoms are commonly managed with preservative‐free (PF) artificial tears, while moderate to severe cases and corneal complications may require topical corticosteroids and cyclosporine/tacrolimus. Severe corneal AEs related to ICIs may require additional autologous serum, vitamin C, oral doxycycline, and discontinuation of ICIs in refractory cases. In cases of corneal perforation, corneal gluing, AMT, corneal crosslinking, or keratoplasty may be required. Corneal graft rejection during ICI therapy can often be managed with corticosteroids but may recur with ongoing therapy. Periorbital edema associated with imatinib may improve with dietary sodium restriction, corticosteroids, and diuretics, though surgical excision of skin and fat may be required in persistent or disfiguring cases. Some cases may progress unfavorably, potentially necessitating modification or discontinuation of oncologic therapy. Early recognition and close collaboration between oncologists and ophthalmologists are crucial to managing these complications while maintaining effective cancer treatment.

## 7 Drug‐Related Blepharoconjunctivitis: Topical Drugs and Preservatives

Topical ophthalmic medications (TOM) are frequently associated with eyelid dermatitis (Figure 4E,F), as the main clinical presentation [128] and, less frequently, with conjunctivitis [129, 130]. Differentiating irritant from allergic contact dermatitis is essential. The natural clearance of allergens by tear flow and the reduced capacity of the OS to develop allergic responses may contribute to the lower prevalence of allergic contact blepharoconjunctivitis when TOM are suspected. Mydriatic agents are the most potent sensitizers, followed by antibiotics, anti‐glaucoma medications, and preservatives (Table S6).

### 7.1 Preservatives in Eyedrops

Preservatives are added to eyedrops to prevent microbial contamination after opening. Preservatives belong to various chemical families including mercury derivatives, alcohols, parabens, EDTA, and chlorhexidine. Quaternary ammonium compounds are mainly used due to low allergenic potential and good safety profiles. Benzalkonium chloride (BAK) is an alkylbenzyl‐dimethylammonium chloride mixture of C12 and C14 chains, commonly used at concentrations ranging from 0.004% to 0.02%. Although BAK is a well‐known irritant, it is rarely recognized as the primary allergen responsible for contact dermatitis [131, 132]. In diseases requiring long‐term treatments such as DED, allergic conjunctivitis, or glaucoma, preservatives may cause significant AEs. Considering the treatment goals, AEs including stinging, burning, irritation, dryness, or less frequently, conjunctivitis, blepharitis, or corneal damage are often underestimated even though they may impair QoL and reduce treatment adherence [133, 134].

### 7.2 Mechanisms of Toxicity

**FIGURE 5:**
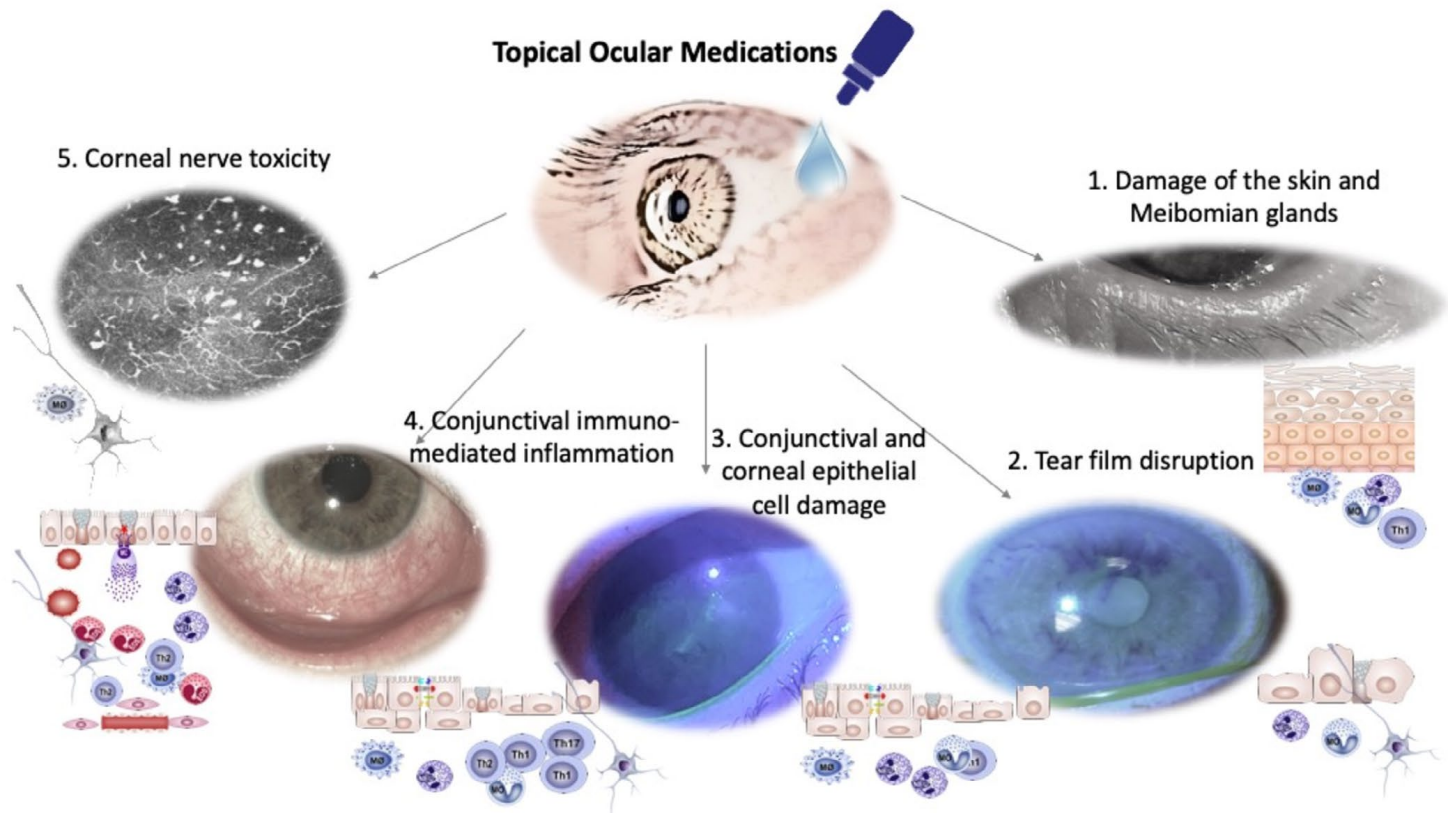
Adverse effects of topical ocular medications (TOM) on the ocular surface. (1) *Damage to the lid skin and meibomian glands*: drug‐induced chronic inflammation stimulates cornified envelope precursors [135], leading to goblet cell entrapment, squamous metaplasia, lid margin keratinization, and further MGD. (2) *Tear film disruption*: goblet cell dysfunction results in the loss of mucin‐related immunosuppressive feedback to dendritic cells, further enhancing chronic inflammation [137]. Tear film dysfunction triggers biological cascades that perpetuate a vicious cycle of neurogenic ocular surface inflammation and further tear film impairment [136]. (3) *Conjunctival and corneal epithelial cell damage*: preservatives and drugs induce a concentration‐dependent decrease in cellular viability, increase apoptosis, and oxidative stress resulting in proinflammatory effects, cytokine release, and increased receptor expression of chemokines and cytokines [138, 139]. (4) *Conjunctival immune‐mediated inflammation*: Th1 and Th2 cytokine profiles are involved, indicating a mixture of allergic and toxic mechanisms [140]. (5) *Corneal nerves neurotoxicity*: preservatives and drugs may also exhibit neurotoxic effects on trigeminal nerve endings [141, 142].

TOM may exert allergic, toxic, or immuno‐inflammatory effects, interacting chemically with different ocular components, inducing: (1) damage to the lid skin and meibomian glands [135]; (2) disruption of the tear film lipid layer, through detergent tensioactive effects, decreased aqueous secretion, and/or goblet cell destruction enhancing chronic inflammation [136, 137]; (3) conjunctival and corneal epithelial cell damage and increased expression of chemokines and cytokines [138, 139, 140]; (4) conjunctival immune‐mediated inflammation through allergic and toxic mechanisms [140]; (5) corneal nerve neurotoxicity [141, 142] (Figure 5).

### 7.3 Anti‐Glaucoma Medications and Antibiotics

Anti‐glaucoma treatments are often administered over decades. While RCTs show relatively good tolerance, observational real‐world studies consistently report a higher incidence of OSD [143, 144]. Approximately 50% of glaucoma patients experience DED symptoms, with 20%–30% of them presenting severe conditions, much higher than the general elderly population (Figure 4E). Allergic blepharoconjunctivitis may also be caused by active pharmaceutical ingredients, such as timolol or brimonidine, whose effects on the OS may interact with the trabecular meshwork and intraocular pressure control [145, 146].

Topical antibiotics are widely used for the treatment of OS infections or perioperative prophylaxis. Aminoglycosides, particularly neomycin, gentamicin, and tobramycin, are most frequently involved in ODHRs [130].

### 7.4 Recommendations for Management

The subtraction strategy (removing the offending agent/s), if possible, is the primary treatment. However, identifying the causative drug can be challenging, especially when: (a) symptoms appear long after treatment initiation; (b) multiple medications are used concurrently; (c) the OS is already impaired; (d) stopping treatment may endanger vision.

Tear substitutes may help to alleviate symptoms with the addition of topical corticosteroids during acute inflammation. Given BAK's dose‐dependent toxicity, reducing the number of preserved eyedrops can reduce AEs [143, 147]. In glaucoma, PF formulations have been developed across multiple drug classes. Laser trabeculoplasty or surgery may be considered when OS health and QoL are severely impaired. Low‐toxicity preservatives (polyquad) are now available, significantly reducing ocular damage [139, 148].

## 8 Cosmetic‐Related Blepharoconjunctivitis

Cosmetics are widely used throughout the world and frequently cause AEs such as allergic contact dermatitis [149], particularly in women [150]. Occupational exposure is a major risk factor. The incidence of contact dermatitis varies between countries, influenced by product availability and differing regulations. In the EU, cosmetic regulation follows the precautionary principle, requiring pre‐market safety assessments and restrictions based on hazard profiles. In the USA, regulation under MOCRA relies more on post‐market surveillance and adverse event reporting to trigger regulatory action [149, 151, 152].

### 8.1 Mechanisms and Clinical Manifestations

Cosmetics can lead to frequent AEs induced by physical trauma, chemical irritation or toxicity, infections, or disruption of the tear film, often leading to OSD [149, 153]. Eye cosmetics commonly cause eyelid contact dermatitis and are frequently associated with blepharoconjunctivitis, which may have an immune‐mediated basis. Eyelid contact dermatitis is more frequently reported than conjunctivitis, with responsible allergens different from those affecting other skin areas [154, 155]. Common allergens include metals (nickel), fragrances, and preservatives, particularly methylisothiazolinone (MI) and methylchloroisothiazolinone, which have shown rising sensitization rates (Table 2). This led to the withdrawal and later ban of MI in stay‐on cosmetic products in the EU and a limitation of 15 ppm use concentration in wash‐off products [165]. Fragrances, preservatives, and metals can be found in a variety of cosmetic products (Table S2). Cosmetic procedures, such as eyelid tattooing, eyelash dyeing, blepharon‐pigmentation, and eyelash extensions, have become increasingly popular. Dyes used in eyelash treatments often contain p‐phenylene diamine and black henna, which can trigger ocular allergic reactions and damage. The glue used in eyelash extensions is primarily cyanoacrylate‐based, containing latex and ammonia, and is known to emit high levels of formaldehyde [153, 166].

**TABLE 2:** Most frequently reported contact allergens associated with eyelid dermatitis, the function of each allergen, and most frequently found sources.

Contact allergens	Functions	Main sources	Prevalence range contact allergy (%)	References
*Metals*				
Nickel	Metallic colorants and glitter effect	Jewelry, makeup, makeup applicators, eye cosmetics (eyeshadow, eyeliner) metal nail files and eyelash curlers	7.0–32.2	[154, 155, 156, 157, 158, 159]
Cobalt		Jewelry, eye cosmetics, hair dye	5.1–8.1	[154, 157, 158]
Gold sodium thiosulfate		Jewelry	3.7–14.7^a^	[154, 156]
Potassium dichromate		Eyeshadows	7.5	[158]
*Fragrances/cosmetics* ^b^				
Fragrance mix I and II		Hair products and hair removal products (shampoos); makeup and makeup remover; moisturizers	4.8–16.5	[154, 155, 157, 159]
Hydroperoxides of linalool		Perfumed cosmetics	2.7	[160]
Balsam of Peru		Perfumed cosmetics	3.5–11.9	[154, 155, 161]
Carmine	Red pigment (from the insect *Dactylopius coccus*)	Cosmetics	5.5	[154]
Shellac	Natural resin derived from the *Kerria lacca* insect	Mascara, tattoo ink, cosmetics, eye cosmetics	4.94	[154, 162]
*Surfactant*				
Dimethylaminopropylamine		Shampoo and eye makeup remover	3.3–4.9	[154, 155]
Oleamidopropyl dimethylamine and cocamidopropyl betaine	Foam booster, mildness, and viscosity control	Surfactant derived from coconut oil	4.7	[154]
Thiuram mix			2.4–4.6	[154, 157]
Sorbitan sesquioleate	Emulsifier	Cosmetics	1.2	[155]
*Preservatives* ^c^				
Formaldehyde and formaldehyde releasers	Cosmetic biocide, preservative and antistatic [153]	Nail polish, adhesives, eyelash glues, mascara, eye makeup remover, around eye cream, serum, eye shadow, moisturizer, glitter	8.7	[154]
Methylisothiazolinone and methylchloroisothiazolinone		False eyelash glue, eye cleansing lotion, makeup remover wipes, mascara, moisturizer, serum, hair products, and shampoos	5.5–16.5	[154, 155, 157, 159]
Benzalkonium chloride	Antimicrobial, antistatic agent, cosmetic biocide, preservative, surfactant [153]	Ophthalmic solutions, shampoos, eyeliner, makeup remover, mascara	4.7–5.0	[154, 159]
Thimerosal			4.0–6.2	[154, 156, 157]
*Acrylates*				
Methacrylate and polymethyl methacrylate (PMMA); hydroxyethyl methacrylate (HEMA) and poly(2 hydroxyethyl methacrylate) (PHEMA)	Suspending agent film‐former, adhesive [153, 163]	Nails cosmetics, around‐eye cream, eyelash glue, eyeliner, eyeshadow, glitter, makeup remover, mascara, serum, soft and hard contact lenses	5.9	[159]

^a^ The clinical relevance remains unknown in most cases.

^b^ Reported fragrances/cosmetics are kathon, lyral, Oakmoss, isoeugenol, benzylsalicylate, and hydroxycitronella.

^c^ Other reported preservatives clorphenesin, ethylhexylglycerin, parabens, kathon CG [153], and pentylene glycol [164]; antibiotics (neomycin and bacitracin) are also used as preservatives.

### 8.2 Diagnosis

A detailed patient history is crucial for diagnosis including the recent introduction of new cosmetic products or the renewal of a previously used item. Patch tests (open or closed, depending on the ingredients) may identify the causative allergen(s) [149, 156]. Proper allergen concentration is essential to avoid false positives and false negatives [167, 168]. Where standard patch testing is inconclusive, repeated open application tests and provocative use tests may help assess clinical relevance [169, 170].

### 8.3 Management

Effective treatment requires strict avoidance of the causative agent and, if possible, finding alternatives. Emollients are key in maintaining skin barrier integrity and should be routinely used. If contact dermatitis does not respond to standard treatment, topical corticosteroids should be considered, but prolonged use should be avoided. Topical tacrolimus may be an effective alternative. Systemic corticosteroids should be reserved for short‐term use and avoided whenever possible [171].

## 9 Conclusions

The main clinical, diagnostic characteristics, and management approaches statements for the different ocular drug hypersensitivity reactions reviewed reached final 100% consensus agreement by the TF members in 15/17 of them. Areas of partial disagreement primarily reflected disparities in access to specific treatment options across countries. For instance, while preservative‐free eyedrops are considered the gold standard for long‐term use, their higher cost and limited availability may render them impractical in some healthcare settings (Table 3).

**TABLE 3:** Final statements and task force members' consensus on the recognition and first management steps for drug‐induced periocular and ocular surface disorders.

Topics	Statements	First management steps	Consensus (% agreement)
Systemic medications and ocular surface disorders	Systemic medications can lead to a range of ocular surface disorders, making early detection and management crucial to prevent irreversible damage. Clinicians must remain vigilant when prescribing drugs with known ocular AEs and collaborate with ophthalmologists for ongoing monitoring	Monitoring	100
	Regular screening, patient education, and interdisciplinary teamwork are vital for minimizing the risks of drug‐induced ocular complications, preserving visual health, and ensuring timely intervention for optimal patient outcomes	Screening risk factors Patient education Referral to ophthalmologist if drug is associated with known ocular AE	100
Severe cutaneous adverse reactions and the ocular surface	There is a rising awareness of ocular surface involvement in drug‐related severe cutaneous adverse reactions. A closer engagement of ophthalmologists during the acute phase is crucial for minimizing complications and visual impairment in the chronic phase	Early ophthalmological monitoring during the acute phase 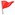	100
	A holistic approach that recognizes critical “intervention windows” and implements timely, effective measures can significantly prevent end‐stage blindness, preserving the patient's quality of life	Ophthalmological management of complications 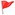	100
Ocular adverse effects of biological treatments	Patients receiving biological therapy, particularly those with likelihood of ocular AEs, should be regularly monitored through ophthalmological evaluations to ensure early detection and management of ocular complications	Regular monitoring of first clinical manifestation	100
	IL‐4/IL‐13 receptor inhibitors for the treatment of atopic dermatitis have increased risk of ocular surface disorders	Start artificial tears in atopic dermatitis patients	100
	When ocular AEs are identified, the addition of topical corticosteroids/immunomodulators and lubricants, discontinuation of the biological agent, and initiation of alternative therapy, may be required	Ophthalmological management of AE 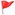	87
Ocular adverse effects in cancer‐targeted therapy and immunotherapy	Cancer immunotherapy, particularly immune checkpoint inhibitors, are associated with ocular AEs, including dry eye and severe corneal complications that may impair vision and quality of life. The risk varies by agent, dosage and duration	Ophthalmologic examination before initiating CTA	100
	Oncologists should inform patients about the potential ocular risks, refer them to the ophthalmologist for baseline eye exams, and consider prophylactic measures such as artificial tears	Patient education Prophylactic artificial tears	100
	At the onset of ocular symptoms, early referral to ophthalmologists is recommended	Early referral to ophthalmologist 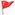	100
	Treatment decisions should balance cancer control with ocular safety, considering whether to continue, adjust, or discontinue therapy based on the severity of eye involvement	Multidisciplinary approach	100
Drug‐related blepharoconjunctivitis: Topical drugs and preservatives	The awareness of sensitivity to topical drugs and preservatives in eyedrops has increased in recent decades	Patient education	100
	Preservative‐free eyedrops are now the gold standard, offering the safest for long‐term use	Preservative free artificial tears (if available)	93
	Single‐dose units and or preservative free multidose bottles are ideal in terms of sterility, although they may pose issues regarding cost, usability (especially disabled patients), and environmental sustainability due to plastic waste	Removing offending agent and avoid preservatives	100
Cosmetic‐related blepharoconjunctivitis	Cosmetics can lead to ocular AEs induced by physical trauma, irritant or toxic effects from chemical constituents, infections, and disruption of the tear film, causing ocular surface disorders	Patient education	100
	Clinical signs and symptoms guide diagnostic work‐up including patch tests with suspected allergens at standardized doses and also with personal cosmetics to confirm sensitization	Multidisciplinary approach	100
	Avoiding the eliciting factor and finding alternatives are the basic managing approaches	Removing all suspected offending agents	100

Red flags = ophthalmic review and input are essential.

Management of ocular drug hypersensitivity requires a multidisciplinary approach. As the range of immune‐modulating therapies continues to expand globally, clinicians must be vigilant about their potential ocular adverse effects. Proactive identification, early ophthalmologic referral, and tailored therapeutic strategies are essential to preserving ocular surface integrity.

## Author Contributions

Conseption and design: A.L. and B.B. Analysis and interpretation: A.L., B.B., L.D., J.‐L.F., S.D., P.D., C.G.M., M.A.‐M., and V.C. Writing the article: A.L., B.B., D.S., C.B., V.S., S.D., S.A., D.P.‐F., M.J.V., F.‐B.B., and G.C. Critical revision of the article: L.D., P.D., C.G.M., M.A.‐M., and J.‐L.F. Final approval of the article: All authors. Data collection: A.L., B.B., D.S., C.B., V.S., S.D., S.A., D.P.‐F., M.J.V., F.‐B.B., G.C., and L.D. Provision of materials, patients, or resources: A.L. and B.B. Statistical expertise: D.S. Obtaining funding: A.L. and B.B. Literature search: A.L., B.B., D.S., C.B., V.S., S.D., S.A., D.P.‐F., M.J.V., F.‐B.B., G.C., and L.D. Administrative, technical, or logistic support: A.L., B.B. and D.S.

## Conflicts of Interest

Andrea Leonardi: Consultancy for Bausch & Lomb, Dompè, FAES Farma, FIDIA, Santen, Théa, SIFI. Christophe Baudouin: Consultancy for Bausch & Lomb, Glaukos, Horus Pharma, Oculis, Santen, and Thea. Vibha Sharma: Consultancy for DBV, Novartis. Jasper Therapeutics. Serge Doan: Received honoraria from Alcon, Almirall, Bausch & Lomb, Horus, Leo Pharma, Sanofi, Santen, Thea. All other authors declare no conflicts of interest.

## Supporting information


**Appendix S1:** all70074‐sup‐0001‐AppendixS1.docx.

**Table S1:** all70074‐sup‐0002‐TablesS1‐S6.docx.
**Table S2:** all70074‐sup‐0002‐TablesS1‐S6.docx.
**Table S3:** all70074‐sup‐0002‐TablesS1‐S6.docx.
**Table S4:** all70074‐sup‐0002‐TablesS1‐S6.docx.
**Table S5:** all70074‐sup‐0002‐TablesS1‐S6.docx.
**Table S6:** all70074‐sup‐0002‐TablesS1‐S6.docx.

## Data Availability

Data sharing not applicable to this article as no datasets were generated or analysed during the current study.
