# Supplementary material for: Drug‐Induced Periocular and Ocular Surface Disorders: An EAACI Position Paper
Source: Allergy. 2025 Sep 30;80(11):2953–72. doi: 10.1111/all.70074 (PMC12590328; doi:10.1111/all.70074)
Supplement: Supplementary file 1 — Appendix S1: all70074‐sup‐0001‐AppendixS1.docx. [file ALL-80-2953-s001.docx]

**Supplementary Methods**

**Systematic search of the literature**

The focus was on the ocular and periocular surface reactions to topical agents and systemic drugs, complemented by the manual search of relevant publications. Where applicable, the keywords used, databases searched, and inclusion and exclusion criteria were specified, particularly in sections supported by more structured evidence or previous systematic reviews. All relevant publications from the past 15 years were reviewed. Each chapter includes an analysis of original studies and narrative and systematic reviews, where available. Data on the most prevalent drugs, allergens, and the products containing those allergens were extracted to be reviewed. Case reports were included if they specifically described associated ocular symptoms. For the different chapters, a systematic search on PubMed Medline, CINAHL and/or Embase databases was performed between August 2024 and May 2025.

*Systemic Medications and Ocular Surface Disorders section*

A systematic search was conducted to include studies reporting associations between systemic medications and ocular surface disorders, focusing on the last five years. Original research articles, case series, and systematic reviews were selected. No language restrictions were applied. Search strategy: ("dry eye" OR "ocular surface disease") AND (toxicity OR "adverse effects") AND ("systemic medications" OR "systemic drugs").

Of 272 studies identified in the last five years, 15 relevant articles were selected, including meta-analyses, randomized controlled trials, and systematic reviews.

*Severe Cutaneous Adverse Reactions (SCARs) and the Ocular Surface section*

A systematic search was conducted in PubMed/MEDLINE to identify studies reporting ocular surface manifestations associated with severe cutaneous adverse reactions (SCARs), including Stevens-Johnson Syndrome (SJS), Toxic Epidermal Necrolysis (TEN), and Drug Reaction with Eosinophilia and Systemic Symptoms (DRESS). No language restrictions were initially applied.

Search strategy: ("Stevens-Johnson Syndrome" OR "Toxic Epidermal Necrolysis" OR "drug-induced skin reactions") AND ("ocular surface" OR "ocular complications" OR conjunctivitis OR keratitis OR "dry eye disease" OR "lid margin keratinization" OR "punctal cautery" OR "mucous membrane graft" OR keratoprosthesis).

Of the 65 studies identified, 17 were included. These included original research articles, case series and comprehensive/ systematic reviews published in peer-reviewed journals that reported on ocular surface involvement either acute or chronic in patients with SCARs. Given the rarity of the disease most were retrospective studies, case series/reports or reviews. Prospective studies on management were few and limited to case series without controls.

*Ocular Adverse Effects of Biological Treatments section*

A literature search was undertaken in MEDLINE, CINAHL, and Embase in August 2024 and updated in May 2025. Publications involving human subjects and written in English were reviewed if they reported associations between biological treatments and ocular adverse effects.

Search strategy: ("TNF-alpha inhibitor" OR "tumor necrosis factor inhibitor" OR adalimumab OR etanercept OR infliximab AND ("ocular surface" OR cornea OR conjunctiva) AND ("adverse effects" OR "side effects" OR toxicity OR "ocular complications"). Of the 155 references found, 24 were chosen, focusing primarily on reported systematic reviews and meta-analysis, clinical trials, retrospective cohort results and mostly case reports.

*Ocular Adverse Effects in Cancer-Targeted Therapy*

A systematic search was conducted in PubMed/MEDLINE to identify studies reporting ocular adverse effects associated with Cancer-Targeted Therapy. The following query and search strategy was used: ("Epidermal growth factor receptor (EGFR) inhibitors, ocular adverse effects"[Mesh] OR "(Tyrosine kinase inhibitors (TKI ), ocular adverse effects" [Title/Abstract] OR ‘’Immune checkpoint inhibitors (ICIs), Ocular adverse effects "[Title/Abstract] OR "CTLA-4 ocular adverse effects" [All Fields]) AND ("PD-1 ocular adverse effects"[MeSH] OR "Lymphocyte activating gene-3, ocular adverse effects"[MeSH] OR ‘’Antibody-drug conjugates (ADC), Ocular adverse effects’’"[Title/Abstract] OR "Cancer-Targeted Therapy, Ocular Adverse effects"[Title/Abstract] OR ‘’Cancer-Targeted Therapy, Conjunctivitis, Dry eye’’ [Title/Abstract]. Of the 169 references found, 61 were selected, including systematic reviews and meta-analysis, clinical trials, retrospective cohort studies, case series and case reports.

*Drug-related Blepharoconjunctivitis section*

A systematic search of the literature was performed using pubmed-medline, all papers from the A systematic search was performed using PubMed/MEDLINE, reviewing all publications from the past 10 years. Due to limited literature reporting on conjunctivitis or blepharoconjunctivitis specifically, all cases of eyelid contact dermatitis associated with topical ophthalmic drugs were included and analyzed. Search strategy: ("Dermatitis, Allergic Contact"[Mesh] OR "Allergic Contact Dermatitis"[Title/Abstract] OR "Contact Dermatitis, Allergic"[Title/Abstract] OR "Allergic Contact Dermatitis" [All Fields]) AND ("Ophthalmic Solutions"[Mesh] OR "Ophthalmic Solutions"[Title/Abstract] OR "Topical Ophthalmic"[Title/Abstract] OR "Eye Drops"[Title/Abstract] OR "Ophthalmic Preparations"[Title/Abstract] OR "Topical Ophthalmic Medications"[Title/Abstract]).

Of the 47 references found, 17 were chosen, focusing primarily on reported large case series/retrospective cohort results. The most important allergens and the prevalence of positivity in patch tests were compiled.

*Cosmetic-related Blepharoconjunctivitis section*

A systematic search of PubMed/MEDLINE was conducted, reviewing all relevant publications from the past 10 years, including narrative and systematic reviews. All allergens associated with cosmetic-related blepharoconjunctivitis or eyelid contact dermatitis were included, given the limited reporting of conjunctivitis symptoms. Search strategy : ("Dermatitis, Allergic Contact"[Mesh] OR "Allergic Contact Dermatitis"[Title/Abstract] OR "Contact Dermatitis, Allergic"[Title/Abstract] OR "Allergic Contact Dermatitis" [All Fields]) AND ("Cosmetic Techniques"[MeSH] OR "Cosmetics"[MeSH] OR "Cosmetics"[Title/Abstract] OR "Cosmetic-Related"[Title/Abstract] OR "Cosmetic Allergy"[Title/Abstract] OR "Eyelash Extensions"[All fields] OR "Eyelid Tattooing"[Title/Abstract] OR "Tattooing"[MeSH] OR "Tattooing"[Title/Abstract] OR "Eyelid Tattoo"[Title/Abstract]) AND ("Eye" OR "Ocular"OR "Eyelid" OR "Periocular" OR "Palpebral" OR "Conjunctivitis").

Of the 44 studies found, 12 were selected. They focused on a case series addressing eyelid dermatitis; case reports were selected if they specifically mentioned associated ocular symptoms. At least two systematic reviews were found, one addressing eyelid contact dermatitis and the other focusing on the ocular surface-related impact. As few studies specifically addressed conjunctivitis or blepharoconjunctivitis, the sources of allergens summarized pertain to eyelid dermatitis.

**Expert consensus**

To classify each position statement, an expert panel was assembled, comprising specialists in the field of ocular allergy and ocular surface disease (OSD), including experts in ophthalmology, allergy, and clinical immunology, as well as researchers and academics with relevant experience. All members formally declared any potential conflicts of interest. A nominal group technique was employed during a virtual meeting, where proposed statements were discussed and refined. Finally, each statement was voted on. Consensus was defined as achieving ≥80% agreement among panel members. Identifying research gaps alongside each position statement aims to guide future studies and facilitate evidence generation in this underexplored field.
