# Supplementary material for: Drug‐Induced Periocular and Ocular Surface Disorders: An EAACI Position Paper
Source: Allergy. 2025 Sep 30;80(11):2953–72. doi: 10.1111/all.70074 (PMC12590328; doi:10.1111/all.70074)
Supplement: Supplementary file 2 — Table S1: all70074‐sup‐0002‐TablesS1‐S6.docx. Table S2: all70074‐sup‐0002‐TablesS1‐S6.docx. Table S3: all70074‐sup‐0002‐TablesS1‐S6.docx. Table S4: all70074‐sup‐0002‐TablesS1‐S6.docx. Table S5: all70074‐sup‐0002‐TablesS1‐S6.docx. Table S6: all70074‐sup‐0002‐TablesS1‐S6.docx. [file ALL-80-2953-s002.docx]

**Supplementary Tables**

**Table 1S. Surgical intervention in SCARs associated ocular surface diseases**

| **Surgical Interventions** | **Indication** | **Rationale** |
| --- | --- | --- |
| **Acute Stage:**  Amniotic membrane transplantation (AMT) | Large areas of epithelial defect | Acts as basement membrane to facilitate epithelization  Growth factors and anti-inflammatory factors further improve the milieu |
| **Chronic Stage:**  Surface stabilization  1.Punctal cautery /plugs  2.Mucous membrane grafting (MMG)  3.Symblepharon release  Visual Rehabilitation:  1.Prosthetic replacement of Ocular surface ecosystem (PROSE)  2.Keratoprosthesis  Modified osteodontokeratoprosthesis (MOOKP)  Boston type 2 Keratoprosthesis | Severe dry eyes  Lid margin keratinization  (Blink related microtrauma)  Exposure, mechanical trauma,  Or visual rehabilitation  To fit scleral lenses  To aid vision  To improve comfort  Dry keratinized surface  Good oral hygiene  Fit for multiple general anesthesia  Dry keratinized surface | To retain the tears and improve the wetness of the eyes  Lid margin keratinization causes blink related microtrauma resulting in punctate keratitis, corneal vascularization, limbal stem cell deficiency and finally affecting vision. Replacing the keratinized tissue with MMG prevents the constant mechanical trauma  Symblepharon bands may cause exposure by causing incomplete lid closure or an inturning of lids and mechanical rubbing on the surface by the lashes and releasing the same helps stabilize the surface.  Symblepharon might prevent a proper fit of scleral lens for vision and need to be released  In scarred keratinized corneas, placing a fluid filled scleral lens improves the surface and aids in improving vision  Canine tooth is used as a carrier for an optical cylinder. The entire complex is anchored in the eye in a 3 staged surgery to aid vision.  A single staged surgery where a polymethylmethacrylate device with donor corneal tissue is anchored in the eye to aid vision. |

**Table 2S. Ocular Adverse Effects associated with Drugs According to Common Terminology Criteria for Adverse Events (CTCAE)** *(revised from National Cancer Institute, 2017).* [*https://ctep.cancer.gov/protocoldevelopment/electronic_applications/docs/ctcae_v5_quick_reference_5x7.pdf*](https://ctep.cancer.gov/protocoldevelopment/electronic_applications/docs/ctcae_v5_quick_reference_5x7.pdf)

| **CTCAE Term** | **Grade 1** | **Grade 2** | **Grade 3** | **Grade 4** |
| --- | --- | --- | --- | --- |
| **Conjunctivitis** | Asymptomatic / mild symptoms | Symptomatic; moderate decrease in visual acuity (BCVA >20/40 or 3 lines or less decreased vision from known baseline) | Symptomatic with marked decrease in visual acuity (BCVA< 20/40 or more than 3 lines of decreased vision from known baseline, up to 20/200); limiting self-care ADL | BCVA of 20/200 or worse in the affected eye |
| **Dry Eye Disease** | Asymptomatic; clinical or diagnostic observations only. | Symptomatic; moderate decrease in visual acuity (BCVA >20/40 or 3 lines or less decreased vision from known baseline) | Symptomatic with marked decrease in visual acuity (BCVA< 20/40 or more than 3 lines of decreased vision from known baseline, up to 20/200); limiting self-care ADL |  |
| **Keratitis** | Asymptomatic; clinical or diagnostic observations only; intervention not indicated | Symptomatic; moderate decrease in visual acuity (BCVA > 20/40 or 3 lines or less decreased vision from known baseline) | Symptomatic with marked decrease in visual acuity (BCVA < 20/40 or more than 3 lines of decreased vision from known baseline, up to 20/200); corneal ulcer; limiting self-care ADL | Perforation; BCVA of 20/200 or worse in the affected eye |
| **Cornea Ulcer** |  |  | Corneal ulcer without perforation in the affected eye | Perforation in the affected eye |
| **Periorbital edema** | Soft or non-pitting | Indurated or pitting edema; topical intervention indicated | Edema associated with visual disturbance; increased intraocular pressure, glaucoma or retinal hemorrhage; optic neuritis; diuretics indicated; operative intervention indicated |  |
| **Other Eye Disorders** | Asymptomatic or mild symptoms; clinical or diagnostic observations only; intervention not indicated; no change in vision | Moderate; minimal, local or noninvasive intervention indicated; limiting instrumental ADL; BCVA > 20/40 or 3 lines or less decreased vision from known baseline | Severe or medically significant but not immediately sightthreatening; limiting self-care ADL; decrease in visual acuity (BCVA< 20/40 or more than 3 lines of decreased vision from known baseline, up to 20/200) | Sight-threatening consequences; urgent intervention indicated; best corrected visual acuity of 20/200 or worse in the affected eye |

ADL: activities of daily living; BCVA: best-corrected visual acuity

### **Table 3S.** **Ocular Surface Adverse Events of Signal Transduction Inhibitors**

| **Signal Transduction Inhibitor** | **Molecule** | **Therapeutic Indications** | **Reported Ocular Surface AEs** |
| --- | --- | --- | --- |
| **Anti-EGFR mAb** | Cetuximab, panitumumab | Colorectal cancer, squamous cell carcinoma of the head and neck | Trichomegaly, trichiasis, eyebrow hypertrichosis, blepharitis, MGD, DED, conjunctivitis, and keratitis.^1-8^ |
| **EGFR TKI** | Erlotinib, gefitinib, afatinib, osimertinib | Non-small cell lung cancer, pancreatic adenocarcinoma | Trichomegaly, trichiasis, eyebrow hypertrichosis, blepharitis, DED, conjunctivitis, keratitis, delayed corneal epithelial healing, vortex keratopathy.^7-17^ |
| **BCR/ABL, PDGF and c-KIT TKI** | Imatinib | Chronic myeloid leukaemia, Gastrointestinal stromal tumor | Periorbital edema, epiphora, conjunctival hemorrhages.^18-23^ |
| **EGFR, VEGFR, RET proto-oncogene TKI** | Vandetanib | Medullary thyroid cancer | Vortex keratopathy.^15, 24-25^ |
| **FGFRs 1-4 TKI** | Erdafitinib | Metastatic urothelial carcinoma | DED, PUK, Meibomian gland dysfunction.^26^ |
| **P13/AKT, mTOR inh** | Perifosine | Colorectal cancer, Waldenström’s macroglobulinemia, multiple myeloma | Ulcerative keratitis, corneal ring infiltrate.^27-28^ |

EGFR: epidermal growth factor receptor; mAb: monoclonal antibody; VEGFR: vascular endothelial growth factor receptor; TKI: tyrosine kinase inhibitor

MGD: Meibomian gland disfunction; DED: Dry Eye Disease, PUK: peripheral ulcerative keratitis

**References:**

1. Bouche O, Brixi-Benmansour H, Bertin A, Perceau G, Lagarde S. Trichomegaly of the eyelashes following treatment with cetuximab. Ann Oncol. 2005;16(10):1711-2.
2. Cohen PR, Escudier SM, Kurzrock R. Cetuximab-associated elongation of the eyelashes: case report and review of eyelash trichomegaly secondary to epidermal growth factor receptor inhibitors. Am J Clin Dermatol. 2011;12(1):63-7.
3. Bambury R, McCaffrey JA. Trichomegaly of the eyelashes after colorectal cancer treatment with the epidermal growth factor receptor inhibitor cetuximab. Clin Colorectal Cancer. 2009;8(4):235.
4. Rodriguez NA, Ascaso FJ. Trichomegaly and poliosis of the eyelashes during cetuximab treatment of metastatic colorectal cancer. J Clin Oncol. 2011;29(18):e532-3.
5. Koksal UI, Pilanci KN, Ordu C, Okutur K, Saglam S, Demir G. Trichomegaly Induced by Cetuximab: Case Series and Review the Literature. Am J Ther. 2016;23(5):e1226-9.
6. Matos LV, Pissarra A, Malheiro M, Placido AN. Trichomegaly of the eyelashes induced by the epidermal growth factor receptor inhibitor cetuximab in the treatment of metastatic colorectal cancer. BMJ Case Rep. 2019;12(4).
7. Fraunfelder FT, Fraunfelder FW. Trichomegaly and other external eye side effects associated with epidermal growth factor. Cutan Ocul Toxicol. 2012;31(3):195-7.
8. Borkar DS, Lacouture ME, Basti S. Spectrum of ocular toxicities from epidermal growth factor receptor inhibitors and their intermediate-term follow-up: a five-year review. Support Care Cancer. 2013;21(4):1167-74.
9. Carser JE, Summers YJ. Trichomegaly of the eyelashes after treatment with erlotinib in non-small cell lung cancer. J Thorac Oncol. 2006;1(9):1040-1.
10. Lane K, Goldstein SM. Erlotinib-associated trichomegaly. Ophthalmic Plast Reconstr Surg. 2007;23(1):65-6.
11. Braiteh F, Kurzrock R, Johnson FM. Trichomegaly of the eyelashes after lung cancer treatment with the epidermal growth factor receptor inhibitor erlotinib. J Clin Oncol. 2008;26(20):3460-2.
12. Pascual JC, Banuls J, Belinchon I, Blanes M, Massuti B. Trichomegaly following treatment with gefitinib (ZD1839). Br J Dermatol. 2004;151(5):1111-2.
13. Joganathan V, Norris JH. Periocular Manifestations of Afatinib Therapy. Ophthalmic Plast Reconstr Surg. 2019;35(1):e12-e3.
14. Paul T, Schumann C, Rudiger S, Boeck S, Heinemann V, Kachele V, et al. Cytokine regulation by epidermal growth factor receptor inhibitors and epidermal growth factor receptor inhibitor associated skin toxicity in cancer patients. Eur J Cancer. 2014;50(11):1855-63.
15. Shin E, Lim DH, Han J, Nam DH, Park K, Ahn MJ, et al. Markedly increased ocular side effect causing severe vision deterioration after chemotherapy using new or investigational epidermal or fibroblast growth factor receptor inhibitors. BMC Ophthalmol. 2020;20(1):19.
16. Li M, Xiang J, Zhang C. When EGFR inhibitor meets autoimmune disease: Severe corneal complications in a patient with Sjogren syndrome after erlotinib treatment. Eur J Ophthalmol. 2022;32(1):NP31-NP4.
17. Johnson KS, Levin F, Chu DS. Persistent corneal epithelial defect associated with erlotinib treatment. Cornea. 2009 Jul;28(6):706-7.
18. Esmaeli B, Diba R, Ahmadi MA, Saadati HG, Faustina MM, Shepler TR, et al. Periorbital oedema and epiphora as ocular side effects of imatinib mesylate (Gleevec). Eye (Lond). 2004;18(7):760-2.
19. Dogan SS, Esmaeli B. Ocular side effects associated with imatinib mesylate and perifosine for gastrointestinal stromal tumor. Hematol Oncol Clin North Am. 2009;23(1):109-14, ix.
20. Fraunfelder FW, Solomon J, Druker BJ, Esmaeli B, Kuyl J. Ocular side-effects associated with imatinib mesylate (Gleevec). J Ocul Pharmacol Ther. 2003;19(4):371-5.
21. McClelland CM, Harocopos GJ, Custer PL. Periorbital edema secondary to imatinib mesylate. Clin Ophthalmol. 2010;4:427-31.
22. Pietras K, Ostman A, Sjoquist M, Buchdunger E, Reed RK, Heldin CH, et al. Inhibition of platelet-derived growth factor receptors reduces interstitial hypertension and increases transcapillary transport in tumors. Cancer Res. 2001;61(7):2929-34.
23. Radaelli F, Vener C, Ripamonti F, Iurlo A, Colombi M, Artoni A, et al. Conjunctival hemorrhagic events associated with imatinib mesylate. Int J Hematol. 2007;86(5):390-3.
24. Ahn J, Wee WR, Lee JH, Hyon JY. Vortex keratopathy in a patient receiving vandetanib for non-small cell lung cancer. *Korean J Ophthalmol*. 2011;25:355–357.
25. Arriola-Villalobos P, Benito-Pascual B, Díaz-Valle D, Benítez-Del-Castillo JM. Confocal microscopy observation of cornea verticillata after vandetanib therapy for medullary thyroid carcinoma. *Cornea*. 2018;37:789–792.
26. Kayabaşı M, Bilkay Görken İ, Durak İ, Karaoğlu A, Saatci AO. Erdafitinib-Induced Bilateral Multifocal Serous Retinal Detachments and Severe Dry Eye Related Unilateral Peripheral Ulcerative Keratitis in a Patient with Metastatic Urothelial Carcinoma. Eur J Case Rep Intern Med. 2024 May 20;11(6):004556.
27. Shome D, Trent J, Espandar L, Hatef E, Araujo DM, Song CD, et al. Ulcerative keratitis in gastrointestinal stromal tumor patients treated with perifosine. Ophthalmology. 2008;115(3):483-7.
28. Keenan JD, Fram NR, McLeod SD, Strauss EC, Margolis TP. Perifosine-related rapidly progressive corneal ring infiltrate. Cornea. 2010;29(5):583-5.

**Table 4S. Ocular Surface Adverse Events of Checkpoint Inhibitors**

| **Immune Checkpoint Inhibitor** | **Target** | **Therapeutic Indication** | **Reported Ocular Surface Adverse Events** |
| --- | --- | --- | --- |
| **Pembrolizumab** | anti-PD-1 | Urothelial cancer, breast cancer, cervical cancer, colorectal cancer, endometrial cancer, esophageal cancer, head and neck epidermoid carcinoma, renal cell carcinoma, NSCLC, classical Hodgkin lymphoma, melanoma, gastric cancer | DED, Conjunctivitis ^1-4^ corneal ulceration and perforation^5-6^, corneal graft rejection^7^ |
| **Nivolumab** | anti-PD-1 | Urothelial cancer, colorectal cancer, esophageal cancer, head and neck cancer, renal cell carcinoma, NSCLC, classical Hodgkin lymphoma, melanoma, malignant mesothelioma, gastric cancer | DED^1,3,8^ corneal ulceration and perforation^8^, ulcerative keratitis^9-10^ |
| **Atezolizumab Durvalumab Avelumab** | anti-PD-L1 | Urothelial cancer, liver and bile duct cancer, NSCLC, melanoma, soft tissue sarcoma, Merkel cell carcinoma | DED^1-3,11^, conjunctivitis and keratitis^11^, corneal perforation^12^ |
| **Ipilimumab** | anti-CTLA-4 | Colorectal cancer, esophageal cancer, renal cell carcinoma, NSCLC, malignant mesothelioma, melanoma | Granulomatous inflammation of lacrimal gland and DED^13^, conjunctivitis, peripheral ulcerative keratitis and corneal perforation^10, 14^ |
| **Tobemstomig/RO7247669** | PD1-LAG3 bispecific antibody | Melanoma | DED, Corneal melting and perforation^15^ |

DED: Dry Eye Disease; NSCLC:

**References:**

1. Zhou L, Wei X. Ocular Immune-Related Adverse Events Associated With Immune Checkpoint Inhibitors in Lung Cancer. Frontiers in immunology. 2021;12:701951.
2. Fortes BH, Liou H, Dalvin LA. Ophthalmic adverse effects of immune checkpoint inhibitors: the Mayo Clinic experience. Br J Ophthalmol. 2021 Sep;105(9):1263-1271,
3. Ramos-Casals M,. Sicca/Sjögren's syndrome triggered by PD-1/PD-L1 checkpoint inhibitors. Data from the International ImmunoCancer Registry (ICIR). Clin Exp Rheumatol. 2019 May-Jun;37 Suppl 118(3):114-122.
4. Young L, Finnigan S, Streicher H, Chen HX, Murray J, Sen HN, Sharon E. Ocular adverse events in PD-1 and PD-L1 inhibitors. J Immunother Cancer. 2021 Jul;9(7):e002119.
5. Weng CC, Wu CC, Lin PY. Corneal melting in a case undergoing treatment with pembrolizumab. Clin Exp Optom. 2020;103(3):379-81.
6. Ramaekers A, Aspeslagh S, De Brucker N, Van Mierlo C, Ten Tusscher M, Schauwvlieghe PP, et al. Bilateral Corneal Perforation in a Patient Under Anti-PD1 Therapy. Cornea. 2021;40(2):245-7.
7. Vanhonsebrouck E, Van De Walle M, Lybaert W, Kruse V, Roels D. Bilateral Corneal Graft Rejection Associated With Pembrolizumab Treatment. Cornea. 2020;39(11):1436-8.
8. Nguyen AT, Elia M, Materin MA, Sznol M, Chow J. Cyclosporine for Dry Eye Associated With Nivolumab: A Case Progressing to Corneal Perforation. Cornea. 2016;35(3):399-401.
9. Losonczy G, Gijs M, Nuijts R. Nivolumab-Induced Ulcerative Keratitis-A Case Report. Cornea. 2021;40(5):656-8.
10. Aschauer J, Donner R, Lammer J, Schmidinger G. Bilateral corneal perforation in Ipilimumab/Nivolumab - associated peripheral ulcerative keratitis. Am J Ophthalmol Case Rep. 2022;28:101686.
11. Bitton K, Michot JM, Barreau E, Lambotte O, Haigh O, Marabelle A, et al. Prevalence and Clinical Patterns of Ocular Complications Associated With Anti-PD-1/PD-L1 Anticancer Immunotherapy. Am J Ophthalmol. 2019;202:109-17.
12. Alkharashi MS, Al-Essa RS, Otaif W, Algorashi I. Corneal Perforation in a Patient Treated with Atezolizumab-Bevacizumab Combination Therapy for Unresectable Hepatocellular Carcinoma. Am J Case Rep. 2023;24:e940688.
13. Ileana Dumbrava E, Smith V, Alfattal R, El-Naggar AK, Penas-Prado M, Tsimberidou AM. Autoimmune Granulomatous Inflammation of Lacrimal Glands and Axonal Neuritis Following Treatment With Ipilimumab and Radiation Therapy. J Immunother. 2018;41(7):336-9.
14. Papavasileiou E, Prasad S, Freitag SK, Sobrin L, Lobo AM. Ipilimumab-induced Ocular and Orbital Inflammation--A Case Series and Review of the Literature. Ocul Immunol Inflamm. 2016;24(2):140-6.
15. Gonzalez de Los Martires P, Guerrero Perez G, Gangoitia Gorrotxategi N, Garmendia IS, Olazaran Gamboa L, Jimenez Alonso A, et al. Bilateral Paracentral Corneal Melting and Left-Eye Perforation under Tobemstomig Novel Treatment. Case Rep Ophthalmol. 2024;15(1):108-14.

**Table 5S**. **Ocular Surface Adverse Events of Antibody-Drug Conjugates**

| **Monoclonal Antibody / Payload** | **Therapeutic Indication** | **Targeted Antigen** | **Reported Ocular Surface Adverse Events** |
| --- | --- | --- | --- |
| **Depatuxizumab mafodotin (ABT-414)** | Recurrent glioblastoma | EGFR | MECs, corneal nerve alterations^1-2^ |
| **Belantamab / Mafodotin** | Relapsed or refractory multiple myeloma | BCMA | MECs, corneal nerve alterations, DED.^3-8^ |
| **Mirvetuximab / Soravtansine** | Platinum-resistant ovarian cancer | FRα | MECs, DED, conjunctivitis.^9-11^ |
| **Tisotumab / Vedotin** | Recurrent or metastatic cervical cancer | Tissue factor | DED, conjunctivitis, keratitis.^11-13^ |
| **Trastuzumab / Emtansine**  **Trastuzumab/ duocarmazine**  **Trastuzumab/DM1** | HER2-positive metastatic breast cancer | HER2 | MECs, corneal nerve alterations, DED.^14-17^ |
| **Enfortumab vedotin** | Advanced and metastatic urothelial carcinoma | Nectin-4 | DED, conjunctivitis, punctate keratitis, limbal stem cell deficiency, and Meibomian gland dysfunction.^18^ |

MECs: microcyst-like cornea epithelial cysts; DED: Dry Eye Disease

**References**

1. Parrozzani R, Lombardi G, Midena E, Leonardi F, Londei D, Padovan M, et al. Corneal side effects induced by EGFR-inhibitor antibody-drug conjugate ABT-414 in patients with recurrent glioblastoma: a prospective clinical and confocal microscopy study. Ther Adv Med Oncol. 2020;12:1758835920907543.
2. Lee BA, Lee MS, Maltry AC, Hou JH. Clinical and Histological Characterization of Toxic Keratopathy from Depatuxizumab Mafodotin (ABT-414), an Antibody-Drug Conjugate. Cornea. 2021 Sep 1;40(9):1197-1200.
3. Aschauer J, Donner R, Lammer J, Roberts P, Funk M, Agis H, Schmidinger G. Corneal Toxicity Associated With Belantamab Mafodotin Is Not Restricted to the Epithelium: Neuropathy Studied With Confocal Microscopy. Am J Ophthalmol. 2022 Oct;242:116-124.
4. Marquant K, Quinquenel A, Arndt C, Denoyer A. Corneal in vivo confocal microscopy to detect belantamab mafodotin-induced ocular toxicity early and adjust the dose accordingly: a case report. J Hematol Oncol. 2021 Oct 3;14(1):159.
5. Farooq, A.V., Degli Esposti, S., Popat, R. et al. Correction to: Corneal Epithelial Findings in Patients with Multiple Myeloma Treated with Antibody–Drug Conjugate Belantamab Mafodotin in the Pivotal, Randomized, DREAMM-2 Study. Ophthalmol Ther. 2020;**9**:913–915 (2020).
6. Rousseau A, Michot JM, Labetoulle M. Belantamab Mafotodin-induced epithelial keratopathy masquerading myopic surgery. Ophthalmology. 2020;127:1626.
7. Mohan M, Rein LE, Thalambedu N, et al. Corneal toxicity with belantamab mafodotin: multi-institutional real-life experience. Am J Hematol. 2022;97: E451–e453.
8. Lonial S, Nooka AK, Thulasi P, Badros AZ, Jeng BH, Callander NS, Potter HA, Sborov D, Zaugg BE, Popat R, Degli Esposti S, Byrne J, Opalinska J, Baron J, Piontek T, Gupta I, Dana R, Farooq AV, Colby K, Jakubowiak A. Management of belantamab mafodotin-associated corneal events in patients with relapsed or refractory multiple myeloma (RRMM). Blood Cancer J. 2021 May 26;11(5):103.
9. Corbelli E, Miserocchi E, Marchese A, et al. Ocular toxicity of mirvetuximab. Cornea. 2019;38:229–232.
10. Matulonis UA, Birrer MJ, O’Malley DM, et al. Evaluation of prophylactic corticosteroid eye drop use in the management of corneal abnormalities induced by the antibody-drug conjugate mirvetuximab soravtansine. Clin Cancer Res. 2019;25: 1727–1736.
11. Marshall RF, Xu H, Berkenstock M. Ocular toxicities associated with antibody drug conjugates. Curr Opin Ophthalmol. 2024 Nov 1;35(6):494-498.
12. de Bono JS, Concin N, Hong DS, et al. Tisotumab vedotin in patients with advanced or metastatic solid tumours (InnovaTV 201): a first-in-human, multicentre, phase 1- 2 trial. Lancet Oncol. 2019;20:383–393.
13. Coleman RL, Lorusso D, Gennigens C, et al. Efficacy and safety of tisotumab vedotin in previously treated recurrent or metastatic cervical cancer (innovaTV 204/GOG- 3023/ENGOT-cx6): a multicentre, open-label, single-arm, phase 2 study. Lancet Oncol. 2021;22:609–619.
14. Burris HA, 3rd, Rugo HS, Vukelja SJ, Vogel CL, Borson RA, Limentani S, et al. Phase II study of the antibody drug conjugate trastuzumab-DM1 for the treatment of human epidermal growth factor receptor 2 (HER2)-positive breast cancer after prior HER2-directed therapy. J Clin Oncol. 2011;29(4):398-405).
15. Tsuda M, Takano Y, Shigeyasu C, Imoto S, Yamada M. Abnormal Corneal Lesions Induced by Trastuzumab Emtansine: An Antibody-Drug Conjugate for Breast Cancer. Cornea. 2016 Oct;35(10):1378-80.
16. Banerji U, van Herpen CML, Saura C, Thistlethwaite F, Lord S, Moreno V, Macpherson IR, Boni V, Rolfo C, de Vries EGE, Rottey S, Geenen J, Eskens F, Gil-Martin M, Mommers EC, Koper NP, Aftimos P. Trastuzumab duocarmazine in locally advanced and metastatic solid tumours and HER2-expressing breast cancer: a phase 1 dose-escalation and dose-expansion study. Lancet Oncol. 2019 Aug;20(8):1124-1135.
17. Deklerck E, Denys H, Kreps EO. Corneal features in trastuzumab emtansine treatment: not a rare occurrence. Breast Cancer Res Treat. 2019 Jun;175(2):525-530.
18. Powles T, Rosenberg JE, Sonpavde GP, Loriot Y, Durán I, Lee JL, Matsubara N, Vulsteke C, Castellano D, Wu C, Campbell M, Matsangou M, Petrylak DP. Enfortumab Vedotin in Previously Treated Advanced Urothelial Carcinoma. N Engl J Med. 2021 Mar 25;384(12):1125-1135.

.

**Table 6S. Description of the most frequently found allergens in topical ophthalmic medication (TOM) that induce eyelid contact dermatitis, and the most recent case series that studied the patch test results for ophthalmic medications in the last 10 years**

|  | **Case series**  **Author (year), country** | | | | | **Case reports** |
| --- | --- | --- | --- | --- | --- | --- |
|  | Patch results (n. positive/n. TOM tested or n. clinically relevant/n. positive/n. tested TOM) | | | | |  |
| **Topical ophthalmic medications (TOM)/allergens** | **Svendsen et al (2024), Denmark(1)** | **Ozkaya et al. (2023), Turkey (2)** | **Alves et al (2022), Portugal(3)** | **Ahlström et al. (2022) Denmark(4)** | **Gilissen et al. (2019), Belgium(5)** |  |
| **Antibiotics** |  |  |  |  |  |  |
| Gentamycin |  | 2/10/19 | 2/7/65 |  | 10/274 |  |
| Neomycin |  | 11/17/26 | 5/11/65 |  | 19/15712 |  |
| Tobramycin | 46/200 | 9/9/13 |  |  | 27/198 |  |
| Bacitracin | 2/630 | 2/2/7 | 0/1/65 |  |  |  |
| Chloramphenicol | 13/637 |  | 1/2/65 | 1/1/245 | 12/561 |  |
| Polymyxin B | 4/607 | 1/1/7 | 0/3/65 |  | 12/561 |  |
| Fusidic Acid | - |  | 0/1/65 |  |  |  |
| Nitrofurazone | - | 1/1/2 | - |  |  |  |
| **Anaesthetics** |  |  |  |  |  |  |
| Caine mix II |  |  | 0/4/65 |  |  |  |
| Tetracaine | 1/246 |  | 0/3/65 |  | 1/107 |  |
| Benzocaine |  |  | 0/2/65 |  |  |  |
| Oxybuprocaine | 2/461 |  | 1/1/65 |  |  |  |
| Lidocaine |  |  |  |  |  |  |
| **Corticosteroids** |  |  |  |  |  |  |
| Hydrocortisone; |  |  |  |  | 9/15659 |  |
| Budesonide, tixocortol, dexamethasone, prednisolone |  |  |  |  |  | **(6)** |
| Dexamethasone |  |  |  | 0/1/244 | 6/744 |  |
| **Sympathomimetics/Mydriatics and Cycloplegics** |  |  |  |  |  |  |
| Phenylephrine | 192/604 |  | 1/1/65 | 22/25/247 | 7/55 | **(7, 8)** |
| Atropine | 4/607 |  | 1/1/65 |  | 5/39 |  |
| Tropicamide minims | 4/300 |  |  | 1/1/244 |  |  |
| **Anti-glaucoma medications** |  |  |  |  |  |  |
| Timolol extract or in eye drops | 12/634 | 2/2/2 | 9/10/65 | 8/13/242 |  | **(6)** |
| Betaxolol |  |  | 0/1/65 |  |  |  |
| Carteolol |  |  | 2/3/65 |  |  |  |
| Betaxolol chloridrate |  |  | 0/1/65 |  |  |  |
| Dorzolamide |  |  | 2/2/65 | 3/7/243 |  | **(11)** |
| Brimonidine |  |  |  |  |  | **(12)** |
| Bimatoprost | 2/385 |  | 1/1/65 |  |  |  |
| Latanoprost | 3/385 |  | 1/1/65 | 1//243 |  |  |
| Ripasudil |  |  |  |  |  | **(9, 10)** |
| **Anti-histamines** |  |  |  |  |  |  |
| Alcaftadine |  |  |  |  |  | **(6, 13)** |
| Ketotifen |  |  |  | 5/5/242 | 1/16 | **(14, 15)** |
| **Nonsteroidal anti-inflammatory drugs** |  |  |  |  |  |  |
| Diclofenac |  |  |  |  |  | **(15)** |

References

1. Svendsen SV, Mortz CG, Mose KF. Contact allergy to topical ophthalmic medications: A retrospective single-centre study of three decades. Contact Dermatitis. 2024;91(2):119-25.

2. Ozkaya E, Keskinkaya Z, Babuna Kobaner G. Tobramycin and antiglaucoma agents as increasing culprits of periorbital allergic contact dermatitis from topical ophthalmic medications: A 24-year study from Turkey. Contact Dermatitis. 2023;89(1):37-45.

3. Alves PB, Figueiredo AC, Codeco C, Regateiro FS, Goncalo M. A closer look at allergic contact dermatitis caused by topical ophthalmic medications. Contact Dermatitis. 2022;87(4):331-5.

4. Ahlstrom MG, Skov L, Heegaard S, Zachariae C, Garvey LH, Johansen JD. Topical eye medications causing allergic contact dermatitis. Contact Dermatitis. 2023;88(4):294-9.

5. Gilissen L, De Decker L, Hulshagen T, Goossens A. Allergic contact dermatitis caused by topical ophthalmic medications: Keep an eye on it! Contact Dermatitis. 2019;80(5):291-7.

6. Chan YK, Novalo Goto ES, Fachini Jardim Criado R, Criado PR. Allergic contact dermatitis by ophthalmological medications in Brazil: experience of a dermatology department. Eur Ann Allergy Clin Immunol. 2021;53(6):280-3.

7. Lokhande AJ, Soni R, D'Souza P, Yadav Y, Goel R. Allergic contact dermatitis to phenylephrine eye drops in an infant. Pediatr Dermatol. 2019;36(6):975-7.

8. Kato M, Nitta K, Kano Y, Yamada M, Ishii N, Hashimoto T, et al. Case of phenylephrine hydrochloride-induced periorbital contact dermatitis with fulminant keratoconjunctivitis causing pseudomembrane formation. J Dermatol. 2018;45(2):e27-e8.

9. Kusakabe M, Imai Y, Natsuaki M, Yamanishi K. Allergic Contact Dermatitis Due to Ripasudil Hydrochloride Hydrate in Eye-drops: A Case Report. Acta Derm Venereol. 2018;98(2):278-9.

10. Sotozono A, Arakawa Y, Tamagawa-Mineoka R, Masuda K, Katoh N. Allergic contact dermatitis due to ripasudil in eye drops. Contact Dermatitis. 2021;85(3):379-80.

11. Mitsuyama S, Abe F, Higuchi T. Allergic contact dermatitis due to dorzolamide eyedrops. Contact Dermatitis. 2021;84(1):58-9.

12. Napolitano M, Potestio L, Castagliola C, Fabbrocini G, Patruno C. Allergic contact dermatitis probably due to brimonidine tartrate in eyedrops. Contact Dermatitis. 2021;85(3):382-4.

13. Kim JH, Kim HJ, Kim SW. Allergic contact dermatitis of both eyes caused by alcaftadine 0.25%: a case report. BMC Ophthalmol. 2019;19(1):158.

14. Romita P, Stingeni L, Barlusconi C, Hansel K, Foti C. Allergic contact dermatitis in response to ketotifen fumarate contained in eye drops. Contact Dermatitis. 2020;83(1):35-7.

15. Pegalajar-Garcia MD, Coronel-Gonzalez BA, Navarro-Trivino FJ. [Translated article] Allergic Contact Dermatitis to Topical Ophthalmic Drugs: Review of Frequently Used Allergens in Spain. Actas Dermosifiliogr. 2024;115(6):T572-T82.
